# Potentiation of rifampin activity in a mouse model of tuberculosis by activation of host transcription factor EB

**DOI:** 10.1371/journal.ppat.1008567

**Published:** 2020-06-23

**Authors:** Ruslana Bryk, Shashirekha Mundhra, Xiuju Jiang, Madeleine Wood, Daniel Pfau, Elaina Weber, Suna Park, Li Zhang, Colin Wilson, Renier Van der Westhuyzen, Leslie Street, Kelly Chibale, Matthew Zimmerman, Véronique Dartois, Nunzia Pastore, Andrea Ballabio, Natalie Hawryluk, Stacie Canan, Vikram Khetani, Joseph Camardo, Carl Nathan

**Affiliations:** 1 Department of Microbiology and Immunology, Weill Cornell Medicine, New York, New York, United States of America; 2 Immunology and Microbial Pathogenesis Program, Weill Cornell Graduate School of Medical Sciences, New York, New York, United States of America; 3 Drug Discovery and Development Centre, H3D, University of Cape Town, Rondebosch, South Africa; 4 South African Medical Research Council Drug Discovery and Development Research Unit, Department of Chemistry and Institute of Infectious Disease and Molecular Medicine, University of Cape Town, Rondebosch, South Africa; 5 Center for Discovery and Innovation, Hackensack Meridian Health, Nutley, New Jersey, United States of America; 6 Department of Molecular and Human Genetics, Baylor College of Medicine, Houston, Texas, United States of America; 7 Telethon Institute of Genetics and Medicine (TIGEM), Naples, Italy; 8 Medical Genetics, Department of Medical and Translational Sciences, Federico II University, Naples, Italy; 9 Ian and Dan Duncan Neurological Research Institute, Texas Children Hospital, Houston, Texas, United States of America; 10 Celgene Global Health, San Diego, California, United States of America; 11 Celgene Global Health, Summit, New Jersey, United States of America; McGill UniversityHealth Centre, CANADA

## Abstract

Efforts at host-directed therapy of tuberculosis have produced little control of the disease in experimental animals to date. This is not surprising, given that few specific host targets have been validated, and reciprocally, many of the compounds tested potentially impact multiple targets with both beneficial and detrimental consequences. This puts a premium on identifying appropriate molecular targets and subjecting them to more selective modulation. We discovered an aminopyrimidine small molecule, 2062, that had no direct antimycobacterial activity, but synergized with rifampin to reduce bacterial burden in Mtb infected macrophages and mice and also dampened lung immunopathology. We used 2062 and its inactive congeners as tool compounds to identify host targets. By biochemical, pharmacologic, transcriptomic and genetic approaches, we found that 2062’s beneficial effects on Mtb control and clearance in macrophages and in mice are associated with activation of transcription factor EB via an organellar stress response. 2062-dependent TFEB activation led to improved autophagy, lysosomal acidification and lysosomal degradation, promoting bacterial clearance in macrophages. Deletion of TFEB resulted in the loss of IFNγ-dependent control of Mtb replication in macrophages. 2062 also targeted multiple kinases, such as PIKfyve, VPS34, JAKs and Tyk2, whose inhibition likely limited 2062’s efficacy in vivo. These findings support a search for selective activators of TFEB for HDT of TB.

## Author summary

Tuberculosis (TB) is now the leading cause of death from a single infection. Effective TB treatments take months, are toxic and lead to the development of multidrug- (MDR) and extensively drug-resistant (XDR) TB. Host-directed therapies (HDT) are being explored for their potential to enhance the efficacy of anti-mycobacterial agents and reduce lung pathology. Rifampin is one of the most important TB drugs but many patients taking the standard dose have suboptimal plasma levels. We discovered an aminopyrimidine compound with no direct antimycobacterial activity that enhanced control of *Mycobacterium tuberculosis* (Mtb) in combination with low-dose rifampin in macrophages and in mice. Beneficial effects correlated with the activation of the host transcription factor EB, a master regulator of lysosomal biogenesis and lysosomal activation. Selective activators of host TFEB may aid in the management of TB in patients with suboptimal plasma levels of rifampin.

## Introduction

TB remains a global health emergency despite intensive research into new ways of combating the disease. Current treatment regimens for drug sensitive TB involve 4 drugs (rifampin (R), isoniazid (H), pyrazinamide (Z), and ethambutol (E)) for two months with two of them (RH) for another 4 months. Treatments are lengthy, often toxic, and, with the spread of drug resistance, increasingly ineffective. Clinical cure often leaves patients with ongoing inflammation [1] and irreversible pulmonary pathology. The search for safer, faster-acting drugs that inhibit new targets in Mtb is being complemented by an effort to find effective HDT. HDT has the ambitious goals of enhancing the immune response and not just avoiding exacerbation of immunopathology but reducing it. Sustaining the search for HDT is an underlying premise: that immunopathology may scale linearly with bacterial burden, so that a HDT may help preserve lung structure and function even if the effect on colony-forming units (CFU) is considered modest by the log-scale criteria conventionally used to evaluate direct-acting antimicrobial agents.

Rifampin remains the cornerstone of TB chemotherapy since its discovery over five decades ago. Plasma levels of rifampin during treatment are known to be highly heterogeneous in the TB patient population, varying as much as 100-fold, and may depend on a patient’s genetics, age, gender, and co-infection status with HIV [2]. The standard daily dose of rifampin of 600 mg in TB patients, which was chosen decades ago, lies on the lower side of the effective dosing range [3]. Patients with suboptimal rifampin plasma levels may be among those who could benefit from adjunctive HDT.

To date the evidence is limited that any host-targeting agent reproducibly reduces CFU of Mtb in otherwise genetically and pharmacologically unmanipulated animals infected with virulent Mtb when the HDT is administered after the onset of disease and as the sole intervention. The current portfolio of candidates for HDT of TB [4–7] includes candidates that were nominated based on treating mice infected with mycobacteria of a less virulent form than wild type Mtb [8,9], treating before the onset of disease [8,10], treating after knock out of a key element of immunity [11], or dosing with another agent that deviates the immune response [11], while others are effective against some Mtb strains and not others [12]. In some cases, the reduction in Mtb CFU was significant only after censoring some results [13], amounted to only ~0.2 log_10_ [14,15], was reported from single experiments [13,15], or was not independently reproducible [16,17]. Nonetheless, collectively, these studies stimulate interest in adjunctive HDT of TB.

The successful development of HDT will likely depend on selective targeting of identified pathways. Screens with siRNA or small molecule libraries in human or mouse macrophages have pioneered the field and are among the approaches that have identified potential HDT targets [14,18,19]. These include cAMP phosphodiesterases, AMP kinase, Akt kinase, Abl kinase, PPARs, matrix metalloproteinases, 3-hydroxy-3-methyl-glutaryl-coenzyme A reductase, and sirtuins [20]. Most studies have used drugs that have known mechanisms of action in the treatment of non-infectious diseases. It has generally been assumed that inhibition or activation of the same targets acting in the same pathways accounts for their impact in TB, but this may not be the case. As a whole, the results with strikingly diverse agents can be interpreted as evidence for many independent pathways. However, it is also possible that some of these routes converge on a common pathway whose selective perturbation could benefit the host with TB.

Given this background, we set out to select a tool compound with a beneficial effect on host control of Mtb alone or in combination with rifampin, identify as many of its targets as possible, and distinguish which of them afford benefit when perturbed. We identified such a compound, an aminopyrimidine numbered 2062, and found that it enhanced the antimycobacterial effect of rifampin in Mtb-infected macrophages and reduced the burden of Mtb in mice also in combination with rifampin. As with many bioactive compounds, the aminopyrimidine proved to have multiple targets. Many of its bioactive cellular targets included kinases whose inhibition would not benefit a host with TB. However, improved control of Mtb in macrophages and in mice in combination with rifampin correlated with the activation of the host transcription factor EB (TFEB), which promotes lysosomal activation to enhance bacterial clearance. Our findings point to selective activation of TFEB as a goal for the development of the next generation of candidates for HDT of TB, which can be used in combination with rifampin in vivo.

## Results

### Identification of small molecule modulators of macrophage activation

**Fig 1:**
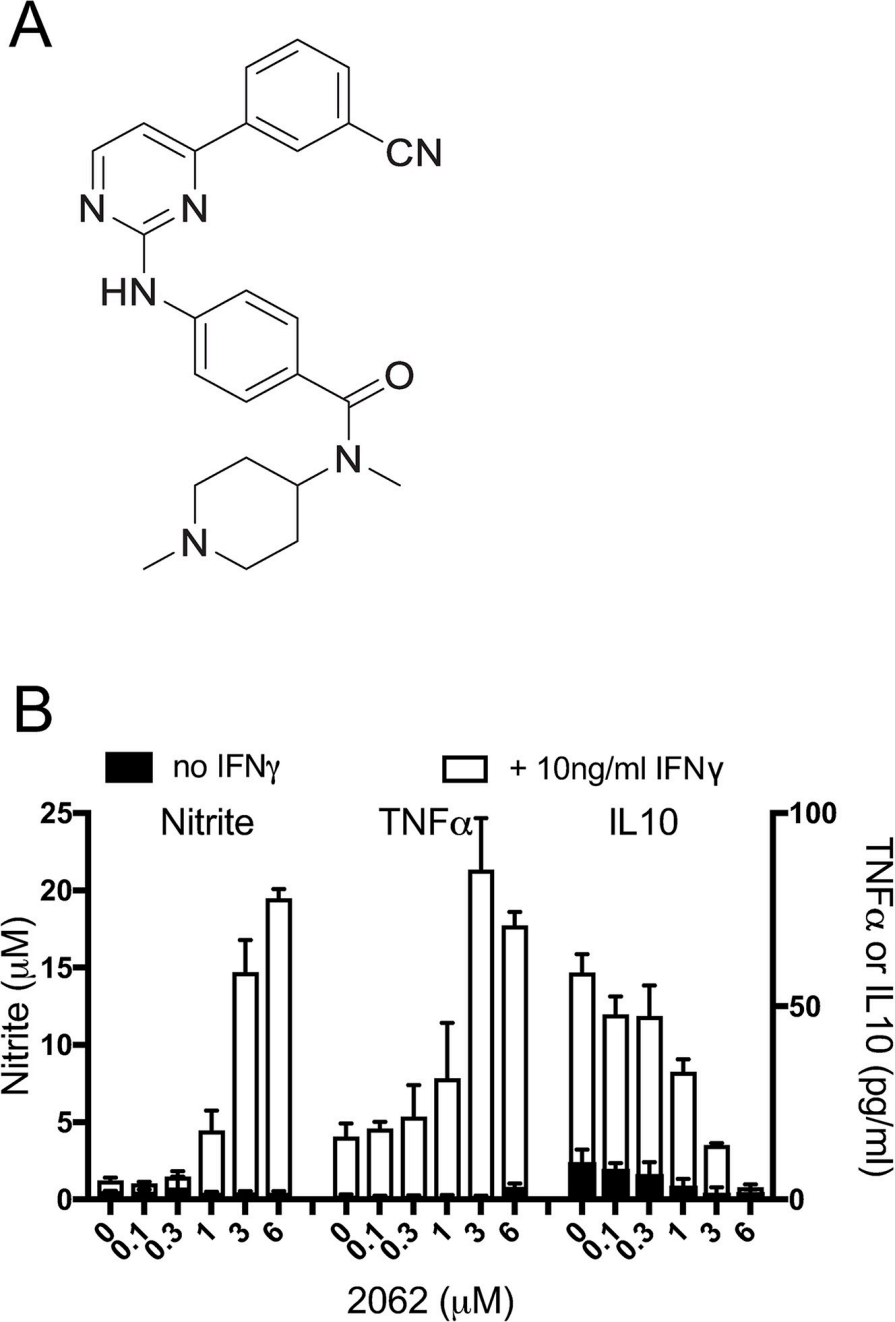
2062 modulates macrophage activation in vitro. **(A)** Structure of 2062. **(B)** Impact of 2062 on activation of BMDM that were exposed or not to IFNγ. BMDM were treated with indicated concentrations of 2062 in triplicate wells without (black bars) or with 10 ng/mL IFNγ (white bars). Media were collected 48 h later and tested for nitrite by Greiss assay and levels of TNFα, and IL10 determined by ELISA. Results are means ± SD of triplicate wells in a single experiment representative of at least 3 independent experiments.

We screened over 2000 compounds representing a diverse library of small molecules from Celgene Corporation for their ability to enhance the production of nitric oxide (measured as nitrite, one of its oxidation products) and tumor necrosis factor α (TNFα) from C57BL/6 mouse bone marrow-derived macrophages (BMDM) in the presence of interferon γ (IFNγ). Several compounds of the 2-aminopyrimidine class, such as 2062 (Fig 1A), markedly boosted nitrite and TNFα and suppressed IFNγ-dependent release of the Th1-suppressing cytokine IL10 in a dose-dependent manner (Fig 1B). After exploring the structure-activity relationship (SAR) and property space of 160 close analogs, we selected 2062 for further study based on its potency, selectivity and favorable absorption, distribution, metabolism, excretion (ADME) properties.

**Fig 2:**
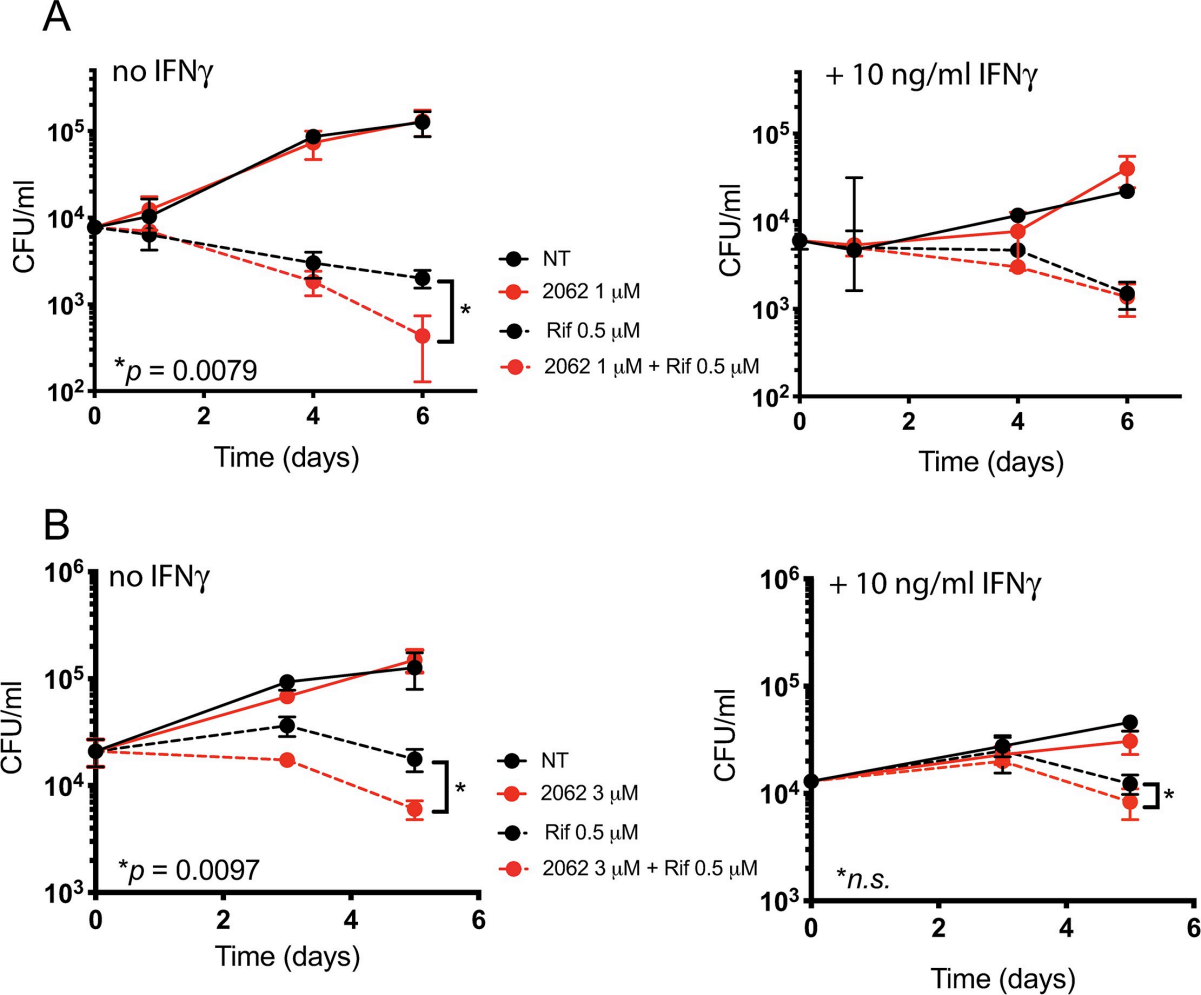
2062 and rifampin reduce bacterial burden in Mtb-infected macrophages. BMDM exposed or not to IFNγ (10 ng/mL) were infected with Mtb H37Rv at MOI of 0.1 for 4 hours, washed, and left untreated (solid black lines) or treated with 2062 alone (solid red lines), rifampin alone (dashed black lines) or the combination of 2062 and rifampin (dashed red lines). **(A)** 2062 was used at 1 μM and rifampin at 0.5 μM. **(B)** 2062 was used at 3 μM and rifampin at 0.5 μM. Cells were lysed at the indicated times for determination of CFU. Results are mean ± SD of triplicate wells in a single experiment representative of 3 independent experiments. P values were calculated by unpaired t-test, n.s., not significant.

Since 2062 enhanced the production of nitric oxide (NO) and TNFα by macrophages, we tested if 2062 would reduce the burden of intracellular bacteria in Mtb-infected BMDM. 2062 had no effect on Mtb in BMDM when tested alone, but enhanced the ability of a suboptimal dose of rifampin (0.5 μM) to reduce intracellular CFU independently of IFNγ (Fig 2A and 2B). Similar results were observed when suboptimal isoniazid (INH) was tested instead of rifampin (S1A Fig). The loss of Mtb viability inside the BMDM was not due to cytotoxicity of 2062 or the combination of 2062 plus rifampin to macrophages, as no significant difference in BMDM viability was observed in control and treatment wells at the end of a 6 day treatment period and 2062 was not cytotoxic to other mammalian cells by the MTS assay (S1B Fig). Similarly, 2062 had no effect on the viability of Mtb in broth culture at concentrations up to 100 μM (S1C Fig), nor did it potentiate rifampin’s anti-mycobacterial activity against Mtb in broth culture (S1C Fig). Thus, the action of 2062 on Mtb in BMDM was likely mediated via targets in the host cells.

### Activity of 2062 against Mtb in mice

**Fig 3:**
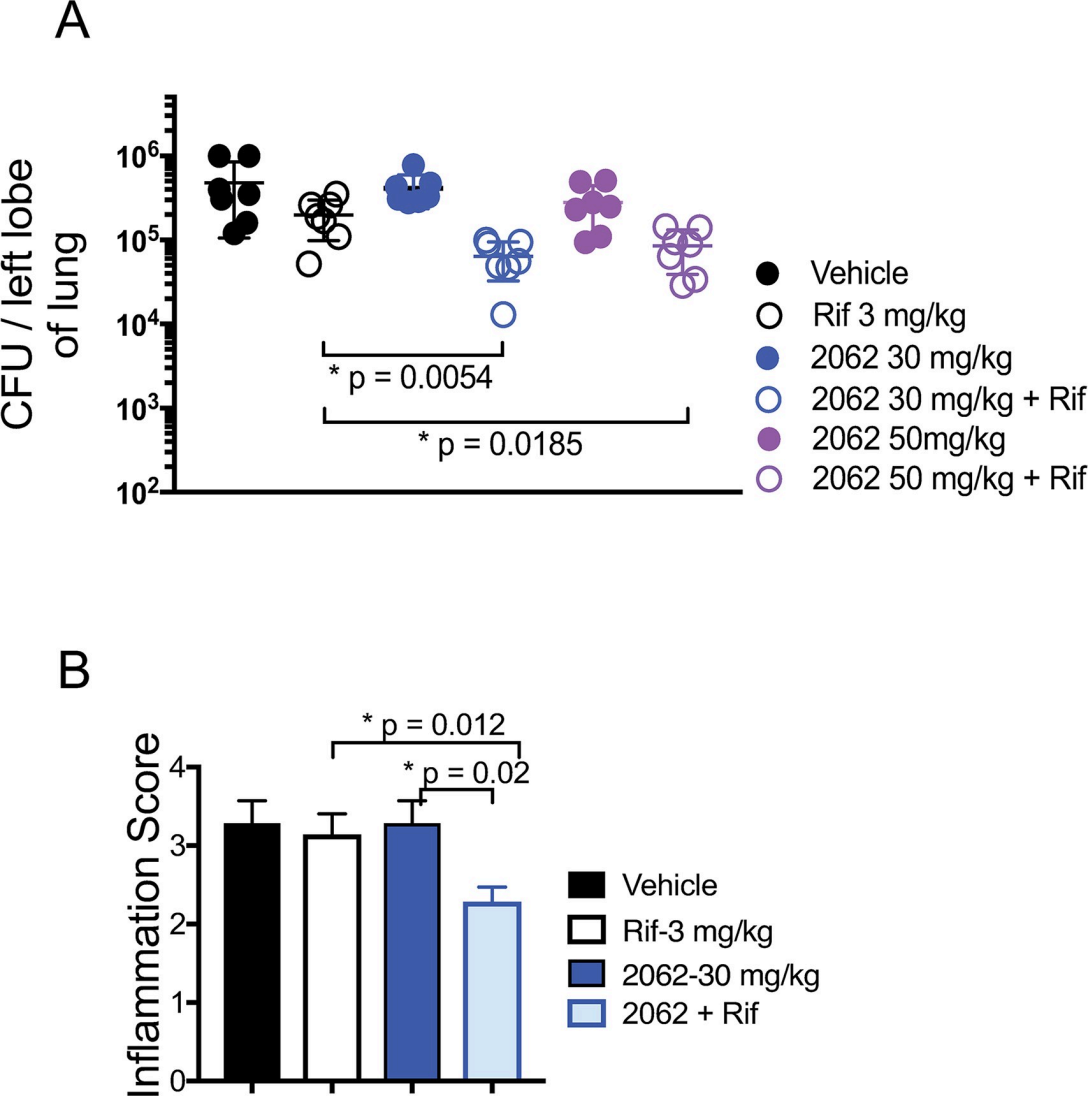
2062 and rifampin reduce the bacterial burden and histopathology in Mtb-infected mice. Mice were infected with Mtb by inhalation and disease was allowed to develop for 2 weeks. **(A)** Treatment was by oral gavage for 6 weeks beginning on day 15, followed by plating of lungs for CFU. Mice were treated with vehicle alone (solid black circles); with 2062 alone at 30 mg/kg (solid blue circles) or 50 mg/kg (solid purple circles); or with rifampin at 3 mg/kg (open circles) given alone or together with the indicated doses of 2062. P values calculated by the unpaired t-test. **(B)** Immunopathology scores of lung slices from mice in **(A)** treated with 30 mg/kg 2062 alone or in combination with 3 mg/kg rifampin. Score numbers reflect the extend of inflammatory lesions: 0, no lesions; 1, minimal inflammation; 2, mild inflammation; 3, moderate inflammation; 4, marked inflammation.

We turned next to *in vivo* experiments in mice. Compound 2062 was well tolerated, as judged by lack of effect on body weight, motility, hemogram, blood chemistries (S2 and S3 Figs) or histology of any organ. However, compound exposure in individual mice varied considerably after intraperitoneal (IP) or oral (PO) dosing (S4 Fig). Also variable were the results in 4 independent experiments in C57BL/6 mice infected with Mtb H37Rv via low-dose aerosol inhalation. The mice were treated with 2062, rifampin, or their combination beginning on day 15 post-infection. IP administration of 2062 alone in the first experiment led to the reduction in bacterial burden in lungs but with high variability among individual mice (S5A Fig, circles). Oral dosing led to a smaller reduction in lung CFU by 2062 alone (0.2 log_10_), but oral co-administration of 2062 and low dose rifampin (3 mg/kg) enhanced the impact of rifampin, reducing CFU by an additional 0.5 log_10_ (Fig 3A, open circles). Mice given 2062 alone or in combination with rifampin consistently gained weight during these studies, in contrast to mice given vehicle alone or rifampin alone (S5B Fig). In an experiment with a more effective dose of rifampin (10 mg/kg), addition of 2062 afforded marginal benefit. Evaluation of the number and size of inflammatory foci in the lungs by a veterinary pathologist blinded to the treatment groups found that co-treatment with 2062 and rifampin led to a statistically significant reduction in the pathologic score (p = 0.02) as compared to the groups receiving vehicle alone, 2062 alone (30 mg/kg) or rifampin alone (3 mg/kg) (Fig 3B).

In summary, 2062 markedly improved the course of TB in combination with rifampin while significantly reducing lung immunopathology in the same treatment group. Encouraged by those results, we set out to identify cellular targets of 2062, which can afford benefit in a TB infected host and potentiate low dose rifampin activity.

### 2062 directly targets multiple kinases

Four complementary approaches were undertaken to identify targets of 2062: comparison of the early RNA-Seq profiles of macrophages treated either with 2062, an active congener, or an inactive congener; inhibitory or binding activity in kinase selectivity panels; comparison of 2062’s effects with the effects of compounds with high potency and specificity for known targets; and differential capture of proteins by pull-downs with probe compounds based on 2062 or on an inactive congener covalently immobilized on magnetic N-hydroxy-succinimide (NHS) beads through an NH_2_-active PEG3 linker (S5C and S6 Figs).

**Fig 4:**
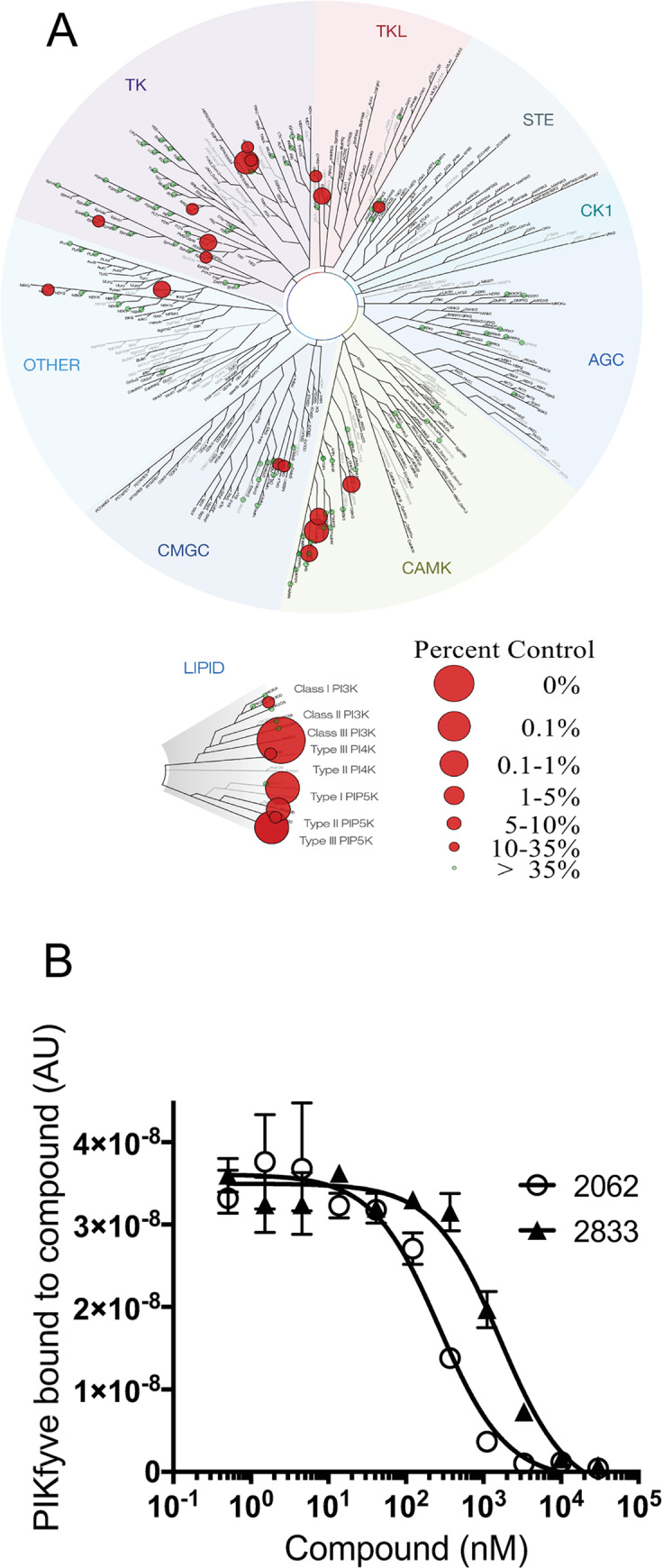
Lipid kinases and PIKfyve are potently inhibited by 2062. **(A)** TREEspot dendrogram depicting the selectivity profile of 2062 (3 μM) tested against 256 human kinases. **(B)** Kd determination for 2062 and related inhibitors binding to PIKfyve by KINOMEscan technology performed at DiscoverX.

The aminopyrimidine core of 2062 suggested that it might be a kinase inhibitor. Indeed, tested at 3 μM, 2062 afforded ≥ 80% inhibition of 31 out of 256 human kinases (Fig 4A and S7 Fig). Targeted profiling against the lipid and AMPK kinase families identified many lipid kinases as high affinity targets, including PIKfyve (Fig 4B), PIP5K1A, PIP5K2B and VPS34. Many of the targets identified through activity profiling or binding studies were among the proteins pulled down from BMDM cell extracts with the active 2062 probe but not with the inactive probe (S6 Fig). Inhibition of many kinases targeted by 2062, such as PIKfyve, VPS34, JAKs and Tyk2, would not benefit a host with TB by impairing endosomal trafficking, phagolysosomal fusion and IFNγ signaling. In fact, inhibition of JAK kinases by 2062 may explain the lower efficacy of 2062 in combination with rifampin in IFNγ-treated BMDM (Fig 2). Thus, we turned to RNA-Seq to further probe cellular networks that became engaged upon 2062 exposure.

### 2062 induces ion imbalance and a stress response

**Fig 5:**
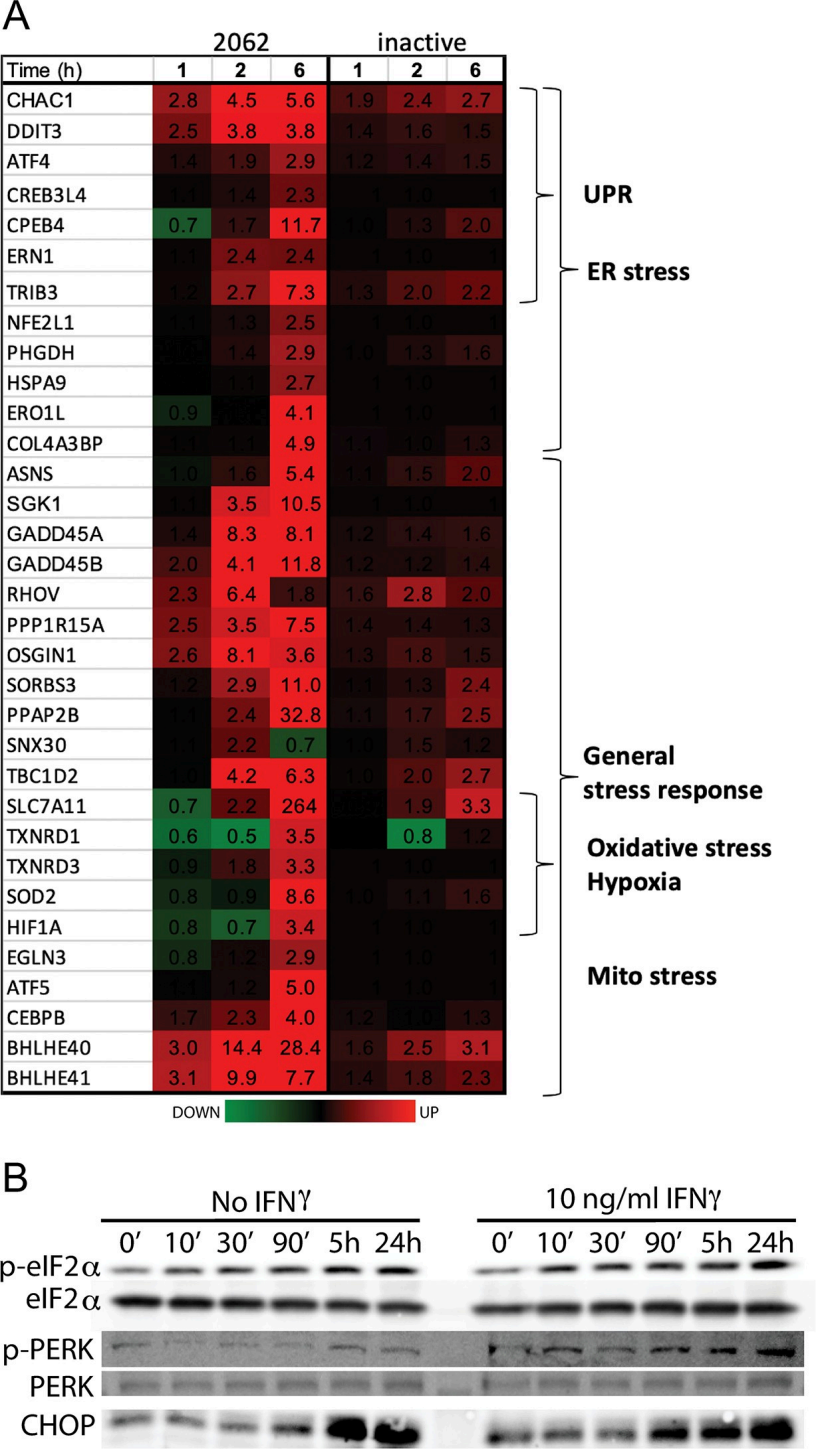
Impact of 2062 on macrophage ER stress response. **(A)** RNA-Seq heatmap of BMDM genes regulated by 2062 (left) or inactive congener (right) over time. Fold change over DMSO control is indicated by numbers. **(B)** 2062 treatment induces a signature response of an ER stress. BMDM were treated for indicated times with 3 μM 2062. Cytosolic fractions separated on 10% SDS-PAGE were probed with antibodies to p-PERK, p-eIF2α and CHOP.

Early changes in the BMDM transcriptome in response to 2062 at 1 and 2 h post treatment indicated an expansive stress response with upregulation of genes involved in the unfolded protein response (UPR) (Fig 5A). Other early-upregulated stress-inducible factors with homeostatic and anti-inflammatory functions included microRNAs (MIR223, MIR25), small nuclear RNAs, and the metabolic, hypoxic and organellar stress response genes GDF15, SIK1, CXCR4, HILPDA, H2-K2 and BHLHE40/41. By 6 h exposure to 2062, many other organellar and oxidative stress response genes became upregulated, along with numerous genes encoding channels and transporters. An inactive congener of 2062 did not elicit these transcriptional changes (Fig 5A).

Consistent with transcriptome changes was the accumulation of signature ER stress response proteins (CHOP) in cell-free extracts prepared from BMDM treated with 2062, along with increased phosphorylation of PERK kinase and its substrate, eIF2α, in a 2062 time- and dose-dependent manner (Fig 5B).

BHLHE40/41 transcription factors have been implicated in the regulation of IL10 production *in vivo* and control of TB in mice [21] and were among the genes upregulated early and most strongly by 2062 (10 μM). However, at the exposure levels of 2062 achieved in BMDM and mice, we saw no substantial changes in protein levels of BHLHE40/41 in 2062-treated macrophages. Moreover, BHLHE40^-/-^ BMDMs and wild type BMDMs produced similar levels of cytokines and nitrite in response to 2062 and showed no difference in their ability to handle Mtb infection with or without 2062.

Extensive dysregulation of ion channels and transporters in response to 2062 suggested possible perturbation of intracellular ions. Indeed, we observed rapid cytosolic alkalinization in BMDMs as measured by fluorescence of the cell-permeable indicator, BCFL-AM inside the cells (S8A Fig) and by real-time extracellular acidification rate (ECAR) measurements in cell culture medium (S8B Fig). Longer exposure to 2062 also perturbed free cytosolic Ca^2+^. Thus, in Ca^2+^-replete medium, 24 h pretreatment with 2062 enhanced the capacitative Ca^2+^ influx through the plasma membrane that was elicited by ionomycin (S8C Fig). In a Ca^2+^-free medium, ionomycin was not able to mobilize Ca^2+^ from intracellular stores in cells pretreated with 2062 for 24 h, consistent with prior 2062-dependent depletion of intracellular Ca^2+^ stores (S8D Fig).

Thus, BMDM response to 2062 featured early upregulation of ER and other organellar stress response genes and intracellular ionic imbalance. Induction of ER stress by 2062 was IFNγ-independent. We suspected that a common mechanism of cellular adaptation to diverse cellular stresses may have benefited Mtb-infected macrophages. Our search for a general stress response mechanism activated upon cellular adaptation to ER stress led us to transcription factor EB (TFEB), which has emerged as a possible mediator of the cellular stress response.

**Fig 6:**
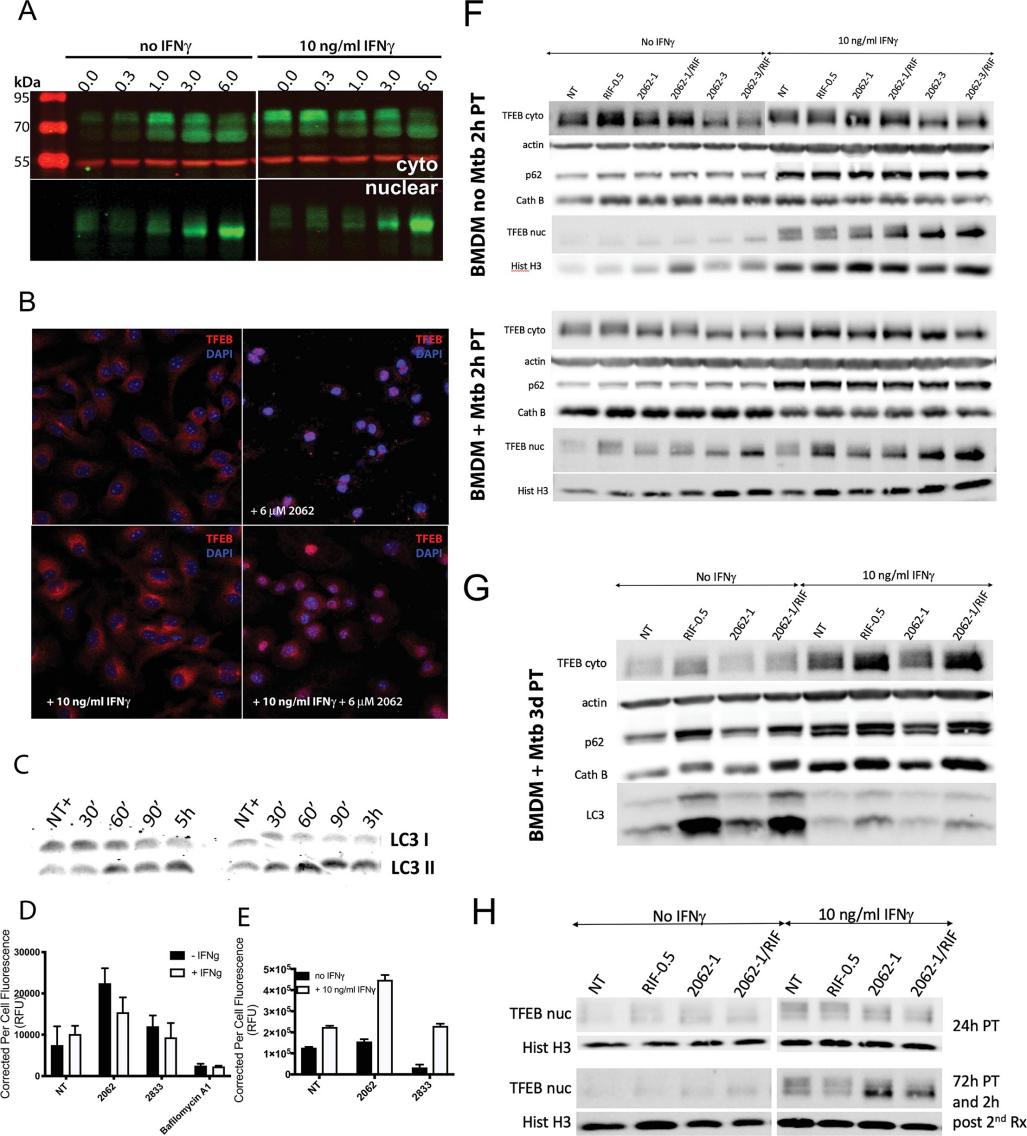
2062 activates TFEB, lysosomal function and autophagy. **(A)** Western blot (WB) of cytoplasmic and nuclear extracts from BMDM treated with concentrations of 2062 indicated in μM terms for 2 h and probed with anti-TFEB (green, 1:1000) or anti-tubulin (red, 1:1000). **(B)** Confocal microscopy of BMDM treated with 2062 for 1 h and stained with anti-TFEB (red) and Hoechst 33342 nuclear stain (blue). **(C)** WB of soluble extracts prepared from BMDM treated with 5 μM 2062 for indicated times and probed with anti-LC3 (1:1000). Shown are two independent experiments. **(D)** LysoTracker fluorescence from BMDM treated with 3 μM 2062, 10 μM 2833, an inactive analog of 2062, and 0.5 μM bafilomycin A1 for 24 h. Each bar represents an average corrected per cell fluorescence from 200–300 cells. Shown is a representative of 3 independent experiments. **(E)** Activity of cathepsin B in BMDM treated with 3 μM 2062 or 10 μM 2833, an inactive analog of 2062, for 24 h. Each bar represents an average corrected per cell fluorescence from 50–100 cells. Shown is a representative of 2 independent experiments. **(F)** BMDM exposed or not to IFNγ (10 ng/ml) and infected or not with Mtb H37Rv (MOI = 0.1) were treated with 2062 at 1 (2062–1) or 3 μM (2062–3), rifampin (0.5 μM, RIF-0.5), or the combination or rifampin plus 2062 (2062-1/RIF; 2062-3/RIF). Cytoplasmic and nuclear extracts were prepared 2 h post treatment. **(G)** BMDM exposed or not to IFNγ (10 ng/ml) and infected with Mtb H37Rv (MOI = 0.1) were treated with 1 μM 2062 (2062–1), rifampin (0.5 μM, RIF-0.5), or the combination or rifampin plus 2062 (2062-1/RIF). Cytoplasmic and nuclear extracts were prepared 3 days post treatment (PT). **(H)** BMDM exposed or not to IFNγ (10 ng/ml) were treated with 1 μM 2062 (2062–1), rifampin (0.5 μM, RIF-0.5), or the combination or rifampin plus 2062 (2062-1/RIF). Two duplicate sets of samples were prepared and initially treated in a similar manner. One set was lysed 24 h post treatment and the second set was left in the incubator for additional 72 h post initial treatment and then re-challenged with the same treatment and extracts prepared 2 h post re-challenge. Top panel shows nuclear extracts prepared 24 h post initial treatment (PT). Bottom panel shows nuclear extracts prepared 2 h after re-challenge of the culture with the same treatments 72 h post initial treatment. Shown is a representative of 2 independent experiments.

TFEB belongs to the family of MiT transcription factors together with MiTF, TFEC and TFE3. TFEB is a master regulator of lysosomal homeostasis and adaptation and is activated upon lysosomal and ER stress [22]. Phospho-TFEB resides in the cytosol in association with mTORC1 at lysosomal membranes. Dephosphorylation of TFEB allows TFEB to translocate to the nucleus and induce the Coordinated Lysosomal Expression and Regulation (CLEAR) regulon, consisting of over 100 genes involved in lysosomal biogenesis and acidification, autophagy, vesicular trafficking and lipid catabolism [22]. We tested whether TFEB is activated upon exposure to 2062 in BMDM by immunoblotting cytosolic and nuclear fractions with anti-TFEB Ab and by confocal microscopy after 1 h treatment with 2062 (Fig 6A and 6B). We observed dephosphorylation of TFEB in the cytosolic fraction and its translocation to the nucleus when BMDM were exposed to ≥ 300 nM 2062. Microscopy confirmed prominent nuclear localization of TFEB at 1 h post-treatment; this was more pronounced in BMDM that were not exposed to IFNγ (Fig 6B). In contrast, activation of MiTF was not observed. TFE3 was already localized in the nucleus without the addition of any of these compounds, perhaps in response to the L-cell conditioned medium used as a source of CSF-1 during differentiation of the BMDM (S9 Fig).

Multiple lines of evidence pointed to the functional impact of TFEB activation. RNA-Seq analysis of genes upregulated after exposure to 2062 in the presence of IFNγ revealed that 11 genes of the CLEAR regulon were upregulated ≥ 2-fold at 2 h and 32 more CLEAR genes were upregulated below 2-fold (S10 Fig). Activation of TFEB promotes autophagy [23]. Immunoblots of cellular extracts showed that exposure to 2062 promoted a time-dependent increase in the lipidated form of LC3, LC3-II (Fig 6C). TFEB activation also promotes lysosomal acidification and the accompanying increase in the degradative capacity of cathepsin B [24]. Consistent with this, LysoTracker fluorescence and the fluorescence activated by cleavage of a cathepsin B substrate both increased in BMDM in the presence of 2062 (Fig 6D and 6E). The 2062-dependent increase in the number of acidic organelles was more pronounced in BMDM that were not exposed to IFNγ, consistent with the higher efficacy of 2062 plus rifampin in Mtb infected BMDM not exposed to IFNγ (Fig 2).

Treatment of Mtb-infected BMDM with 2062 activated TFEB similarly to treatment of uninfected BMDM, with TFEB nuclear translocation observed at 2 h post treatment (Fig 6F). Low dose rifampin promoted greater TFEB activation in IFNγ-activated Mtb-infected macrophages when compared to uninfected BMDM. Single dose treatment with 2062 resulted in transient activation of TFEB with limited nuclear TFEB detected after 24 h (Fig 6G and 6H). The effects of TFEB activation on lysosomal degradation and autophagy were not immediately pronounced in Mtb-infected BMDM upon treatment with 2062 or 2062 plus rifampin (Fig 6F) but became more apparent with longer treatment (Fig 6G and S11 Fig). Thus, at 3 days post treatment a 2062-dependent increase in cathepsin B was detected; it became more pronounced by day 6 (S11B and S11C Fig) in naïve Mtb-infected BMDM but was blunted in IFNγ-activated BMDM. A similar trend in 2062-dependent reduction in p62 levels was detected by ImageJ analysis (S11 Fig). The reduction of p62 also became more pronounced by day 6 post treatment in naïve BMDM. A marked increase in LC3-II was observed in Mtb-infected BMDM treated with rifampin and an additional effect of 2062 was not easily discernible, but 2062 alone led to a detectable increase in LC3-II by day 3 when compared to untreated BMDM (Fig 6G). The effects of 2062, rifampin or their combination on cellular degradation and autophagy pathways were more pronounced in naïve BMDM (Fig 6G and S11 Fig), consistent with the greater loss of Mtb viability inside BMDM treated with 2062 plus rifampin in cells not activated with IFNγ (Fig 2). BMDM exposed to 2062 were able to respond to a second challenge with 2062 or 2062 plus rifampin by activating TFEB again (Fig 6H), suggesting that daily challenge with 2062 may have been able to produce repetitive activation of TFEB *in vivo* during treatment of infected mice (Fig 3).

Three mechanisms are reported to promote TFEB activation — inhibition of mTOR and ERK1/2 [23,25] and activation of calcineurin [26,27]. Kinase selectivity profiling revealed that 2062 does not directly inhibit either mTOR or ERK1/2. However, western blots of BMDM lysates prepared at intervals after exposure of the cells to 2062 demonstrated a dose- and time-dependent inhibition of both mTORC1 and ERK1/2 (S12A Fig). Inhibition of S6K (a readout for mTORC1 inhibition) and ERK1/2 was observed as early as 10 min after exposure of the cells to 2062 at concentrations of 300 nM and above (S12A and S12B Fig). Inhibition of mTOR and ERK1/2 were pronounced with 2062 exposure only in the presence of IFNγ. In contrast, 2062-dependent activation of TFEB was comparable in macrophages with and without IFNγ. This encouraged us to focus on 2062’s activation of calcineurin, which was independent of IFNγ. Calcineurin could be activated as a result of the increase in free cytosolic Ca^2+^ induced by 2062. Application of the calcineurin inhibitor FK506 prevented TFEB’s nuclear translocation at concentrations of 2062 below 1 μM. Chelation of intracellular Ca^2+^ by pretreatment of BMDM with BAPTA-AM completely blocked the ability of 2062 to activate TFEB and activation of NFAT (S12C Fig), a known target of Ca^2+^/calcineurin activation [28]. Moreover, 2062-dependent nuclear translocation of TFEB in BMDM was detected only when the cells were maintained in Ca^2+^-replete medium, either complete DMEM or HBSS. However, when the medium was replaced with HBSS without Ca^2+^, BMDM exposure to 2062 failed to activate TFEB in a dose-dependent manner (S12D Fig), suggesting a role for extracellular Ca^2+^ in 2062-dependent TFEB activation.

### Deletion of TFEB in macrophages impairs control of Mtb

Next, we explored the course of Mtb infection in mouse macrophages lacking TFEB. TFEB deletion is embryonic lethal. However, a mouse line has been generated to delete floxed *Tfeb* alleles in cells expressing Cre driven by a myeloid lysozyme promoter [29]. RAW 264.7 macrophage cell lines that lack TFEB were also generated by the CRISPR-Cas9 method [29]. We compared BMDM from the conditional myeloid cell TFEB knockout mice with the two RAW 264.7 TFEB deletion cell lines, independently produced by the CRISPR-Cas9 method in two different laboratories.

**Fig 7:**
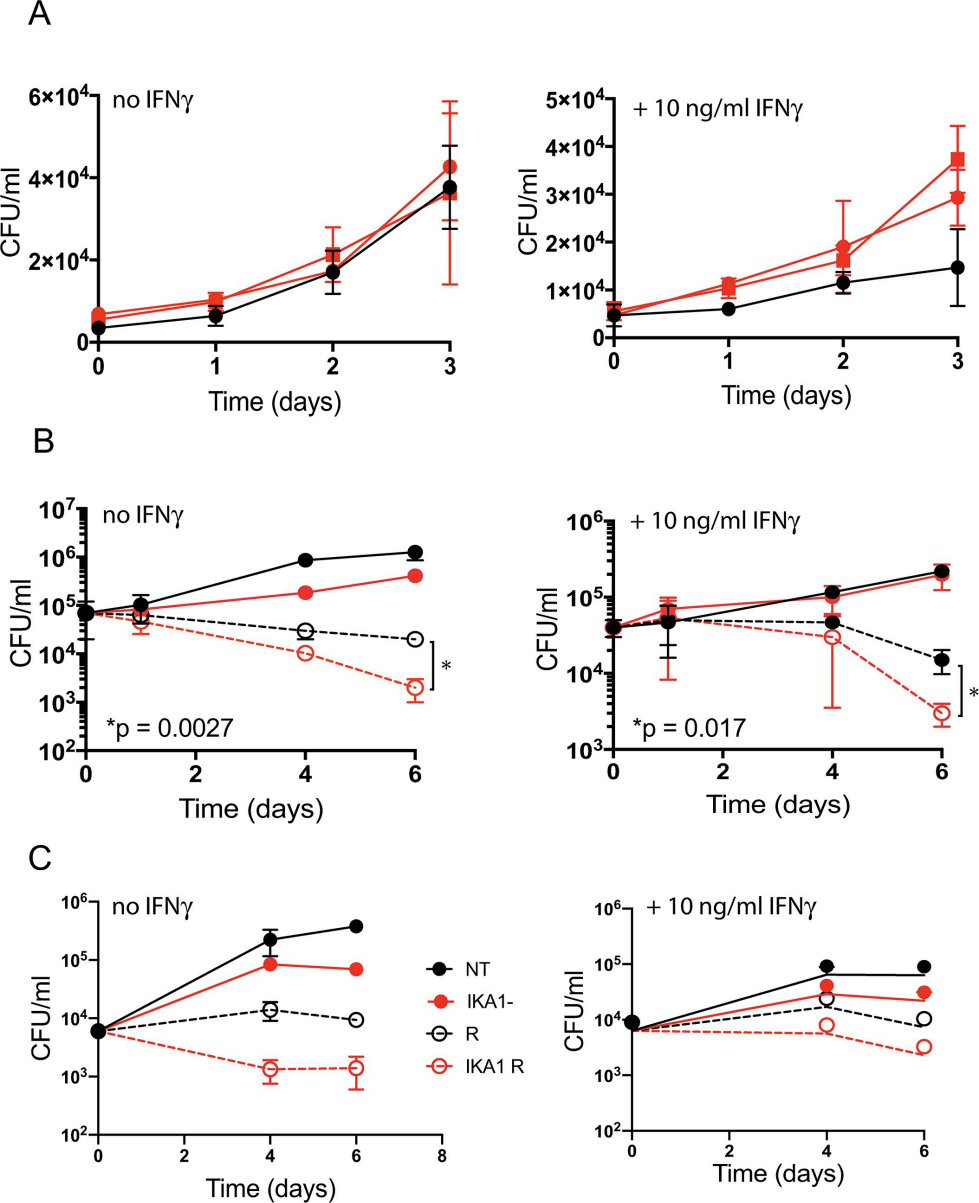
Impact of TFEB gene deletion and Torin 1 (a TFEB activator) on macrophage control of Mtb. **(A)** Loss of control of Mtb growth by activated RAW 264.7 macrophages deleted for *Tfeb* by CRISPR/Cas9. Cells were incubated with IFNγ (10 ng/mL) or not for 24 h before infection with Mtb (MOI = 1) for 4 hours, washed, and lysed at the indicated times for enumeration of CFU. Black, WT macrophages; red, *Tfeb*^-/-^ macrophages from two independently derived lines. **(B)** Enhanced effect of rifampin on CFU in WT Mtb-infected BMDM treated with the TFEB activator Torin 1, mimicking the effect of 2062 seen in Fig 2. BMDM were exposed or not to IFNγ and infected with Mtb as in **(A)** except that the MOI was 0.1 to retain viability of BMDM through the 6-day long experiment, then not further treated (solid black lines) or treated with rifampin alone (0.5 μM; dashed black lines), Torin 1 alone (1 μM, solid red lines) or the combination of rifampin (0.5 μM) and Torin 1 (1 μM) (dashed red lines). Results in **(A)** and **(B)** are means ± SD of three replicate wells representative of 3 independent experiments. Multiple t-test analysis was performed to compare the means and calculate p values in (B). (**C**) BMDM were exposed or not to IFNγ and infected with Mtb as in **(B)** and then not further treated (solid black lines) or treated with rifampin alone (0.5 μM; dashed black lines), ikarugamycin (IKA) alone (1 μM, solid red lines) or the combination of rifampin (0.5 μM) and IKA (1 μM) (dashed red lines). Results are means ± SD of three replicate wells representative of 2 independent experiments.

By immunoblot, no TFEB was detectable in the two independently derived TFEB-deficient RAW 264.7 cell lines. Surprisingly, however, nearly 50% of wild-type levels were observed in BMDM generated in the Nathan laboratory from the femurs of the Lyz2-Cre TFEB^fl/fl^ conditional myeloid knockout mice. This suggests that under the conditions used in the Nathan laboratory, the Cre promoter may have been activated later during *in vitro* macrophage differentiation than the TFEB promoter. At a multiplicity of infection (MOI) = 1, in each of 3 independent experiments, we observed loss of IFNγ-dependent control of Mtb replication in the independently-derived TFEB-deficient RAW 264.7 macrophage lines from the two laboratories (Fig 7A). In contrast, in the primary BMDM population with partial deletion of TFEB, Mtb replicated to a similar level as in the wild- type controls.

We tested if 2062 will potentiate rifampin activity in Mtb infected RAW macrophages deleted for TFEB. In two independent experiments, 2062 alone or in combination with rifampin produced no significantly detectable difference in Mtb control between the WT and TFEB-deficient RAW macrophages (S13 Fig). However, Mtb infection experiments in the RAW macrophages significantly differed from the BMDM infection in the experimental timelines. The effects of 2062 plus rifampin in Mtb-infected BMDM became apparent on days 3–6 post treatment. In contrast, RAW infection experiments lasted only 3 days as macrophage proliferation exhausted the medium.

### TFEB activation promotes macrophage control of Mtb in combination with rifampin

Lastly, we tested the hypothesis that the improved control of Mtb infection in macrophages simultaneously exposed to rifampin and 2062 could be reproduced with a TFEB activator that is more selective than 2062 and does not share 2062’s inhibition of PIKfyve, VPS34, JAKs and Tyk2, among others. Torin 1, an ATP-competitive active site inhibitor of mTOR, was reported to inhibit only 3 out of 442 human kinases at 10 μM [30]. Torin 1 activated TFEB (S14B Fig) similarly to 2062 and likewise had no effect on the growth of Mtb in axenic culture (S14A Fig), but reduced Mtb replication in BMDM without IFNγ. Torin 1 did not enhance IFNγ-dependent control of Mtb in these short-term cultures, but did markedly enhance control of Mtb in the presence of rifampin, whether or not the BMDM were treated with IFNγ (Fig 7B), affording one additional log_10_ reduction in Mtb CFU without IFNγ and nearly 0.7 log_10_ reduction in the presence of IFNγ as compared to rifampin alone.

Recently Wang et al. [31] identified three TFEB pathway agonists in a high throughput screen for TFEB activators. We tested all three compounds (digoxin, alexidine and ikarugamycin) on Mtb-infected BMDM alone and in combination with suboptimal rifampin. At the concentrations reported by Wang et al. [31], digoxin did not have any apparent effect on BMDM control of Mtb (S14C Fig) but neither did it activate TFEB (S14B Fig). Alexidine reduced Mtb loads in BMDM alone and in combination with rifampin (S14C Fig) but it also potently inhibited Mtb replication in broth culture (S14A Fig). Ikarugamycin potently activated TFEB in BMDM (S14B Fig) and reduced Mtb loads in BMDM treated with ikarugamycin alone and in combination with rifampin (Fig 7C). A lower dose of ikarugamycin was not effective in Mtb infected BMDM alone but potentiated low dose rifampin activity similar to 2062 (S14D Fig). Ikarugamycin had no effect on Mtb replication in culture (S14A Fig). Ikarugamycin’s direct targets are unknown and therefore this compound was not tested for its contribution to Mtb control in cell line macrophages lacking functional TFEB.

These findings reinforced the evidence that selective TFEB activators can improve host control of Mtb infection and potentiate low dose rifampin activity.

## Discussion

Host-directed interventions in infectious disease can be a spectacular success when their goal is to induce a normal immune response in advance of infection through vaccination or to restore to normalcy a host immune response crippled by a vitamin deficiency or protein-calorie malnutrition. In contrast, success has been elusive when HDT has been applied after an infectious disease is clinically apparent in a host with a normal immune system. The need for adjunctive HDT is high in TB, given TB’s leading role in infectious disease mortality and the rising resistance to direct-acting antimicrobial therapy. However, TB poses major challenges for HDT. Propagation of TB depends on a vigorous host immune response that generates immunopathology [32]. HDT that boosts the immune response could further harm the host and help spread the pathogen. TB lesions are heterogeneous across space and time in a given host [33], making it impractical to identify patients who would not face this risk. In general, TB HDT agents are not expected to afford substantial reductions in bacterial loads when tested alone in animal models of established TB infection, given that they do not display direct antimicrobial activities. On the other hand, active HDT agents tested in combination with direct-acting antimycobacterial agents may improve host control of TB and limit tissue damage.

Here we report on a small molecule with no direct anti-Mtb activity and with variable pharmacokinetics and efficacy in individual Mtb-infected mice, that afforded improved efficacy in combination with rifampin. We took on the challenge of using the compound as a tool to identify host targets relevant for the control of TB. The compound proved to have multiple targets, each of which is known to exert pleiotropic effects. Along with variable blood levels, the predominance of beneficial effects at lower concentrations and adverse effects at higher concentrations may account for the variation of its impact in individual mice. Tracing the mechanism of action of this compound led us to recognize that activation of TFEB by multiple pathways can account for the beneficial effects observed at lower concentrations.

2062 directly targeted multiple kinases, inhibition of which most likely will not benefit a host with TB. Thus, PIKfyve and its product PI(3,5)P_2_ have been implicated in the regulation of endosomal maturation and trafficking. Inhibition or silencing of PIKfyve in macrophages led to phagosome maturation defects [34] via dysregulation of PI(3,5)P_2_-regulated endosomal Ca^2+^ TRPML1 channels involved in phagolysosomal fusion [35]. 2062 also inhibited Vps34, an endosomal Class III PIPK that generates the initial endosomal PI3P critical for the maturation of phagosomes [36]. The efficacy of 2062 in Mtb-infected mice was achieved at high nanomolar to low micromolar plasma levels, concentrations at which PIKfyve inhibition was not pronounced *in vitro*. Other kinases targeted by 2062 include Jak1/2/3 and Tyk kinases. Inactivating mutations in these kinases are associated with an increased risk of mycobacterial disease [37]. Inhibition of these kinases in Mtb-infected mice treated with 2062 may also have restricted its efficacy. Based on the findings that 2062 targets the above kinases, we do not suggest further progression of the current aminopyrimidine scaffold but instead want to emphasize 2062’s beneficial targets, whose potential may have been masked due to compound’s other pleiotropic effects.

According to our data on diverse inhibitors with high specificity for individual pathways, we hypothesize that 2062’s beneficial effects involve dephosphorylation and activation of TFEB and that this occurs primarily through the induction of an organellar stress response and release of Ca^2+^ from intracellular stores, leading to activation of the phosphatase calcineurin, which dephosphorylates TFEB. Acting indirectly, 2062 also leads to inhibition of mTOR and ERK1/2, kinases that likewise act on TFEB [23,25,26,38].

TFEB activation likely improves control of Mtb in part by the promotion of lysosomal acidification, lysosomal degradative capacity and autophagy. Other effects of TFEB activation have been observed in other settings. Recently discovered TFEB activators improved glucose tolerance and restored insulin sensitivity in mice on a high-fat diet [31]. The three different chemophores that served as TFEB agonists in that study each operated through different targets but converged on perturbation of localized organellar Ca^2+^ fluxes [31]. Over-expression of TFEB with the intent of reducing lysosomal protein loads also appeared to benefit mice with lysosomal storage and neurodegenerative disorders [39–41].

Loss of control over Mtb infection in macrophages lacking TFEB points to the essentiality of TFEB in canonical macrophage activation by IFNγ. This reflects the conservation at the mammalian adaptive-innate immune interface of a process first discovered in the innate immune system of *C*. *elegans*, where expression of 80% of antimicrobial and autophagy genes is controlled by HLH-30, a TFEB homolog. The present findings are also consistent with the role of TFEB in the induction of IL6, IL1β, TNFα, IL27, CCL5 and CCL17 in mouse macrophages [42].

Mtb appears to have developed ways to resist TFEB activation in the host. Mtb was reported to induce the miR-33 locus, thereby repressing autophagy and lysosomal activation and promoting bacterial survival and persistence, while silencing of miR-33/33* activated TFEB, promoted autophagy and enhanced bacterial clearance in macrophages [15]. Likewise, a PPARα -dependent antimycobacterial response operated via activation of TFEB [43]. NR1D1, an orphan nuclear receptor, was implicated in Mtb clearance by inducing lysosomal activation and autophagy via TFEB activation [44]. We observed over 6-fold transcriptional induction of NR1D1 within 2 hours of exposing macrophages to 2062. Finally, metformin was found to inhibit mTORC1 and activate HLH-30, the TFEB homolog in *C*. *elegans* [45], raising the possibility that activation of TFEB may be a shared property of chemically diverse agents that have already been nominated for HDT of TB.

In summary, the findings reported here commend a search for TFEB activators that spare PIKfyve, VPS34, JAKs and Tyks. Compounds with that profile of activity and selectivity would be prime candidates for evaluation as adjunctive host-directed therapeutics in infectious diseases whose causative pathogens persist in host phagolysosomes.

## Methods

### Ethics statement

All animal experiments were performed in accordance with NIH guidelines for housing and care of laboratory animals and WCM institutional regulations after protocol review and approval by the Institutional Animal Care and Use Committee (IACUC Protocols 2013–0001 and 2018–0007).

### Mice

Adult female C57BL/6 mice (Jackson Lab) were aged 6–8 weeks at purchase and rested at least 1 week before use for all experiments.

### BMDM

BMDM were differentiated as reported [46] for 6–7 days in complete DMEM (4.5 g/l glucose, 10% FBS, 1% HEPES, 1% sodium pyruvate, 1% L-glutamine) supplemented with 20% L929 cell-conditioned medium (LCM). Cells were collected in 0.5 mM EDTA in PBS, washed with 10% LCM in complete DMEM, counted by hemocytometer and plated in 48-well plates (2.0–2.5 × 10^5^ cells/well) in 0.5 mL 10% LCM in complete DMEM. Inhibitors were added next day by replacing the medium with fresh medium containing indicated concentrations of inhibitor. No antibiotics were used at any stage in any experiments, except for rifampin where indicated. All cultures were incubated at 37°C in 5% CO_2_, 95% humidified air.

### Test agents

All commercially available inhibitors (bafilomycin A1, apilimod, Torin 1, thapsigargin, FK506, amiloride, rifampin) were from Sigma or Santa Cruz. MF4 and MF2 were kindly provided by Kevan Shokat, UCSF. All other compounds were provided by Celgene Corporation or synthesized at the Drug Discovery and Development Centre (H3D) at the University of Cape Town, South Africa. Inhibitors were prepared as 10 mM stocks in DMSO and tested at 10 μM final concentration (0.1% final DMSO). Where indicated, pure recombinant mouse IFNγ (final 10 ng/mL) (Roche) was added to BMDM cultures either together with inhibitors or 24 h prior to inhibitor treatment. Where indicated, *E*. *coli* LPS (Sigma) was added at 10 ng/mL. Each concentration of each agent was tested in triplicate in each experiment along with non-treated or vehicle (DMSO) treated controls. BMDM were observed daily. Forty-eight hours after addition of inhibitors, a 50 μL aliquot from each well was tested for nitrite and the rest frozen for ELISAs. Cellular viability was tested by the MTS assay (Abcam) according to the manufacturer’s instructions.

### Compound synthesis

Structures of active (2062) and inactive (2833) compounds and their ^1^H NMR features are provided in S15 Fig. Purification of compounds was carried out by either column chromatography on silica gel 60 (Fluka), particle size 0.063−0.2 mm (70−230 mesh) or by Waters preparative HPLC using X-bridge C18 5μm column (4.6 x 150 mm); Mobile Phase B: 0.4% acetic acid, 10 mM ammonium acetate in a 9:1 ratio of HPLC grade methanol and Type 1 water; Mobile Phase A: 0.4% acetic acid in 10 mM ammonium acetate in HPLC grade (Type 1) water; flow rate = 15.00 mL/min; detector: photodiode array (PDA). All compounds were characterized by 1H NMR and LC-MS. NMR spectra were recorded on either a Varian Mercury-300 (1H 300.1 MHz, 13C 75.5 MHz) or Bruker-400 (1H 400.2 MHz, 13C 100.6 MHz) instrument. Liquid chromatograph with mass spectrometer (LC-MS) analysis was performed using an Agilent 1260 Infinity Binary Pump, Agilent 1260 Infinity Diode Array Detector (DAD), Agilent 1290 Infinity Column Compartment, Agilent 1260 Infinity Standard Autosampler, and an Agilent 6120 Quadrupole (Single) mass spectrometer, equipped with APCI and ESI multimode ionization source. Purity was determined by Agilent LC-MS using a Kinetex Core C18 2.6 μm column (50 x 3 mm); Mobile Phase B: 0.4% acetic acid, 10 mM ammonium acetate in a 9:1 ratio of HPLC grade methanol and Type 1 water; Mobile Phase A: 0.4% acetic acid in 10 mM ammonium acetate in HPLC grade (Type 1) water; with flow rate = 0.9 mL/min; detector: diode array (DAD).

### 4-((4-(3-cyanophenyl)pyrimidin-2-yl)amino)-N-methyl-N-(piperidin-4-yl)benzamide-3-(2-chloropyrimidin-4-yl)benzonitrile

0.4 M Na2CO3 solution (176 mL, 7.83 g in 176 ml water) and Pd(dppf)Cl2 (491 mg, 0.671 mmol) was added to a degassed suspension of 2,4-dichloro-pyrimidine (10.0 g, 67.1 mmol) and (3-cyanophenyl)-boronic acid (9.86 g, 67.1 mmol) in toluene/EtOH (9/1; 250 mL). The reaction mixture was heated to 50°C under nitrogen overnight. The reaction mixture was cooled, the solid collected and dried in an oven at 50°C to afford 3-(2-chloropyrimidin-4-yl)benzonitrile (12.0 g, 83%).

### 4-((4-(3-cyanophenyl)pyrimidin-2-yl)amino)benzoic acid

4-aminobenzoic acid (14.61 mmol, 3 g) was added to a solution of 3-(2-chloropyrimidin-4-yl)benzonitrile (13.91 mmol, 3 g) in 2-ethoxyethanol (50 mL) and heated to 150°C overnight. The resulting precipitate was filtered, washed with EtOH and dried to afford 4-((4-(3-cyanophenyl)pyrimidin-2-yl)amino)benzoic acid as a solid (3.5 g, 80%).

### 4-((4-(3-cyanophenyl)pyrimidin-2-yl)amino)-N-methyl-N-(piperidin-4-yl)benzamide

HATU (9.48 mmol, 3.61 g) was added to a solution of 4-((4-(3-cyanophenyl)pyrimidin-2-yl)amino)benzoic acid (6.32 mmol, 2 g) and DIPEA (12.65 mmol, 2.209 mL) in DMF (10mL) and the solution was stirred for 10 mins after which N,1-dimethylpiperidin-4-amine was added and the solution stirred overnight. The resulting precipitate was filtered, washed with H2O (10 mL), EtOH (10 mL) and ether (10 mL) to afford title compound as a solid (1.85 g, 69%). The solid was further purification by recrystallization in hot EtOH. ^1^H NMR (400 MHz, DMSO-d6) δ 9.95 (s, 1H), 8.65 (d, *J* = 5.2 Hz, 1H), 8.60 (td, *J* = 1.7, 0.6 Hz, 1H), 8.50 (ddd, *J* = 8.0, 1.8, 1.1 Hz, 1H), 8.02 (ddd, *J* = 7.7, 1.6, 1.1 Hz, 1H), 7.87 (d, *J* = 8.6 Hz, 2H), 7.79 (d, *J* = 7.8 Hz, 1H), 7.57 (d, *J* = 5.2 Hz, 1H), 7.36 (d, *J* = 8.5 Hz, 2H), 3.03 (d, *J* = 12.1 Hz, 2H), 1.87 (s, 3H), 1.72 (qd, *J* = 12.2, 3.8 Hz, 2H), 1.60 (d, *J* = 11.8 Hz, 2H).

### N-(1-(2-(2-(2-(2-aminoethoxy)ethoxy)ethoxy)ethyl)piperidin-4-yl)-4-((4-(3-cyanophenyl) pyrimidin-2-yl)amino)-N-methylbenzamide

NaI (109 mg, 0.727 mmol) was added to a solution of 5-(tert-butoxycarbonyl)-2,2-dimethyl-4-oxo-3,8,11,14-tetraoxa-5-azahexadecan-16-ylmethane sulfonate [47] (189 mg, 0.400 mmol) dissolved in acetone (3.6 mL) and DMF (1 mL) in a sealed tube. The mixture was heated for 30 min at 60 ^o^C followed by the addition of *N*-(1-(2-(2-(2-(2-aminoethoxy)ethoxy)-ethoxy)ethyl)piperidin-4-yl)-4-((4-(3-cyanophenyl)pyrimidin-2-yl)amino)- *N*-methylbenzamide (150 mg, 0.364 mmol) and K_2_CO_3_ (101 mg, 0.727 mmol). The mixture was heated for an additional 20 h at 60°C. The reaction was diluted with EtOAc (20 mL), washed with water (10 mL) and sat. Na_2_CO_3_ solution (10 mL). After drying the organic layer over sodium sulfate and concentration under reduced pressure the crude product was purified by flash column chromatography (2–5% 2 M NH_3_ in MeOH/DCM). The obtained residue was dissolved in DCM (3 ml) and trifluoroacetic acid (3 ml) was added. After stirring for 1 h at room temperature the mixture was concentrated under reduced pressure. The residue was dissolved in DMSO (2 mL) and purified using prep-HPLC to afford title compound as an oil. LC-MS (ESI): m/z 588.3 (48)^+^. HPLC purity 95%. ^1^H NMR (300 MHz, Chloroform-d) δ 8.54 (d, *J* = 5.2 Hz, 1H), 8.34 (s, 1H), 8.27 (dt, *J* = 8.0, 1.5 Hz, 1H), 7.82–7.67 (m, 3H), 7.66–7.54 (m, 2H), 7.40 (d, *J* = 8.3 Hz, 2H), 7.17 (d, *J* = 5.2 Hz, 1H), 3.80–3.44 (m, 12H), 3.13–2.78 (m, 6H), 2.51 (s, 6H), 2.29–1.82 (m, 4H).

### RNS assay

Griess assay reagent was prepared by mixing equal parts of reagent A (1% [w/v] naphthylethylenediamine) and reagent B (1% [w/v] sulphanilamide in 5% [v/v] phosphoric acid) and prepared fresh from reagents A and B, which were stored separately at 4°C. The mixture (50 μL) was added to 50 μL of the cell-conditioned medium, using 10% LCM in complete DMEM as a control. Nitrite levels were calculated from a standard curve using sodium nitrite at 2-fold dilutions covering the range 0.39–100 μM.

### ELISAs

Levels of TNFα, IL10, IL6, and IFNβ in the cell-conditioned medium were determined by DuoSet ELISA kits from R&D according to the manufacturer’s instructions. Samples from cells treated with LPS or LPS/IFNγ were diluted 1:5 in corresponding media for TNFα ELISA.

### Infection of BMDM

BMDM were plated in 48-well plates (2.0–2.5 × 10^5^ cells/well) in 0.5 mL 10% LCM in complete DMEM. Where indicated, IFNγ (final 10 ng/mL) was added and cells were left to adhere overnight. A single-cell suspension of Mtb H37Rv in PBS with 0.02% tyloxapol was added in 50 μL to achieve MOI of 0.1 or 1. Four h later, the medium was removed, the monolayers washed twice with warm PBS and 0.5 mL fresh 10% LCM in complete DMEM replaced. BMDM were observed daily and photomicrographs recorded. Only wells with intact BMDM monolayers were used for determination of CFU. Test agents were added 24 h after Mtb infection by replacing the medium in wells with BMDM with 10% LCM in complete DMEM containing the indicated final concentrations of inhibitors. Data are presented according to the time after treatment as opposed to the time after infection. At each time point, the medium was removed, monolayers washed with warm PBS and BMDM lysed with 100 μL of 0.5% Triton X100 in PBS. PBS (400 μL) was immediately added to wells to dilute the Triton X100 to 0.1% to minimize any impact on Mtb viability. Samples were serially diluted and 10 μL plated on 7H11 agar plates incubated at 37°C for CFU enumeration 3 weeks later.

### Infection and treatment of mice

Mice were infected with early log phase Mtb H37Rv via aerosol inhalation to achieve ~100 CFU/lung measured 24 h later (day 1) by homogenizing lungs from each of 3 mice and plating on 7H10 agar plates. On day 14 or 21, 5 mice were used to determine the level of CFU at the start of treatment. Test agents were administered daily 7 days per week for 6 weeks by IP injection or oral gavage as indicated to 5–7 mice per group starting on day 15 or 21 post infection. Injections were in a volume of 100 μL in a vehicle consisting of 5% N-methyl-2-pyrrolidone (NMP)/45% polyethylene glycol (PEG) 400/50% of 5% dextrose in water (D5W). Gavage was in a volume of 200 μL in a vehicle consisting of 0.5% carboxymethyl cellulose (CMC)/0.25% Tween 80. 2062 was dosed at 50 mg/kg (IP) or 10, 30, 50 or 75 mg/kg (PO) without or with 3 mg/kg or 10 mg/kg rifampin as noted. Drug suspensions were prepared daily for IP or weekly for oral administration and homogenized in a bath sonicator for 90 min immediately before use. Where stated, tail blood was collected at 0.5 and 2 h after the final dose for measurement of 2062 levels by LC-MS as described below. Mice were euthanized 24 h after the last dose. Lungs were collected for pathology, cytokines and CFU; spleens for CFU; and mediastinal lymph nodes for FACS analysis. Lung pathology was scored based on extent (size and number of foci) of the inflammatory lesions in a blinded manner by a pathologist (0, no lesions; 1, minimal inflammation; 2, mild inflammation; 3, moderate inflammation; 4, marked inflammation).

### LC/MS-MS analytical methods

DMSO stocks of 2062 were serially diluted in 50/50 acetonitrile water and subsequently serially diluted in drug-free CD1 mouse plasma (K_2_EDTA, Bioreclamation IVT, NY) to create standard curves and quality control (QC) spiking solutions. Standards, QCs, controls, and study samples were extracted by combining plasma with acetonitrile/methanol 50/50 containing the internal standard Verapamil. Extracts were vortexed and centrifuged, and the supernatant was transferred for HPLC-MS/MS analysis. LC/MS-MS was performed on a Sciex Applied Biosystems Qtrap 6500+ triple-quadrupole mass spectrometer coupled to a Shimadzu 30ACMP HPLC system, and chromatography was performed on an Agilent Zorbax SB-C8 column (2.1x30 mm; particle size, 3.5μm). Milli-Q deionized water with 0.1% formic acid was used for the aqueous mobile phase and 0.1% formic acid in acetonitrile for the organic mobile phase. Multiple-reaction monitoring of parent/daughter transitions in electrospray positive-ionization mode was used to quantify all molecules. MRM transitions of 427.10/299.20 and 455.40/165.20 were used for 2062 and verapamil, respectively.

### Mtb MIC

MICs were determined in 7H9/0.02% tyloxapol/10% Middlebrook Oleic Albumin Dextrose Catalase (OADC) supplement on Mtb mc^2^6220 Δ*panCD*Δ*lysA* BSL2 strain and Mtb H37Rv with 2-fold serial dilutions of compounds covering a range of 100 nM-100 μM in 96- or 384-well plates. Growth was measured by optical density and compared to vehicle (DMSO) controls. Mtb strain mc^2^6220 Δ*panCD*Δ*lysA* was passaged in Middlebrook 7H9 supplemented with glycerol (0.5%), OADC, tyloxapol (0.02%), casein hydrolysate (CAS) amino acids (0.05%), L-lysine (240 μg/mL), and pantothenate (24 μg/mL).

### MTS assay

Cellular toxicity was tested by MTS assay (Abcam) according to manufacturer’s instructions. Cells were exposed to 0.1, 0.3, 1, 3, 6, and 10 μM 2062 in triplicate or sextuplate wells of the 96 well plate for 48 h. Viability was calculated relative to vehicle treated controls.

### Lysates

Lysates were prepared from BMDM or RAW 264.7 (ATCC TIB-71) macrophages cultured in 10 cm tissue culture dishes with at least 5x10^6^ cells. The medium was removed and the plates were washed 3 times with warm PBS and forcefully tapped over paper towels to remove the remaining liquid. NE-PER Nuclear and Cytoplasmic Extraction Reagents Kit (ThermoFisher) was used to separate lysates into cytosolic and nuclear fractions according to the manufacturer’s instructions. Buffers were supplemented with complete protease inhibitor cocktail (Roche) and phosphatase inhibitors (PhosSTOP, Sigma). 250 μL of lysis buffer from the kit was applied to the monolayers and the cells collected with a scraper. Cells in lysis buffer were collected for processing according to the manufacturer’s instructions. Protein concentrations were determined by DC Protein Assay (Bio-Rad).

### Pull-downs with probe compounds

Probes were constructed by derivatizing 2062 or an inactive congener with three polyethylene glycol (PEG3) linkers bearing a free NH_2_ group, yielding the compounds shown in S5C Fig. The probe compound based on 2062 affected macrophage release of nitrite, TNF and IL10 in the same manner as 2062. The probe compounds were covalently immobilized on NHS FG Magnetic Beads (Nacalai) according to the manufacturer’s instructions. The derivatized beads were stored in 50% methanol until use. BMDM differentiated in 15-cm Petri dishes were primed or not with 10 ng/mL IFNγ for 24 h, washed with warm PBS, lifted from the plates with 1 mM EDTA in PBS, collected by centrifugation, resuspended in 1 mL lysis buffer (50 mM HEPES, pH 7.5, 150 mM NaCl, 0.5% NP40, 2 mM EDTA, 1 mM DTT, 1 mM PMSF, protease inhibitor cocktail), and cooled on ice for 10 min with vortexing every 2 mins. The soluble fraction of the lysates was collected by centrifugation at 10,000 g for 15 min. Protein was measured by DC Protein Assay (Bio-Rad) and diluted to 3 mg/mL with the binding and wash buffer (20 mM HEPES, pH 7.9, 100 mM KCl, 1 mM MgCl_2_, 0.2 mM CaCl_2_, 0.2 mM EDTA, 10% (v/v) glycerol, 0.1% NP40, 1 mM DTT, 0.2 mM PMSF). Derivatized beads were washed 3 times with the same buffer and collected on the magnetic stand. 0.5–1 mg of beads was incubated with lysates (1 mg protein) with rocking at 4°C for 4 h. The beads were then separated on the magnetic stand and washed 3 times with the wash buffer. Bound proteins were eluted by boiling with the Laemmli sample buffer (LSB) or by incubation with the wash buffer containing an excess of 2062 (1 mM) or 1 M KCl. Negative controls included beads without a probe compound, beads with the inactive probe compound and inclusion of an excess of 2062 (1 mM) during incubation with the lysates. Eluted proteins were separated on SDS-PAGE, stained with silver stain or Coomassie and identified by peptide mass fingerprinting. Sets of 3 biological replicates for active and inactive probe compounds were compared using Proteome Discoverer/MASCOT and MaxQUANT/Andromeda to identify and rank statistically significant targets.

### Antibodies

Antibodies against the listed antigens were from the following sources: TFEB (A303-673A, Bethyl Labs), p70 S6 kinase (9202S, CST), phospho-p70 (Thr389) S6 kinase (9205S, CST), p44/42 MAPK (9102S, CST), phospho-p44/42 MAPK (9101S, CST), tubulin (DM1A, Santa Cruz Bio), LC3 (L7543, Sigma), eIF2a (5324S, CST), phospho-eIF2a (Ser51) (ab32157, Abcam), PERK (3192S, CST), phospho-PERK (Thr980) (3179S, CST), CHOP 9C8 (MA1-250, ThermoFisher), NKAα 1 (3010S, CST), actin (G043, abm), histone H3 (9715S, CST), cathepsin B (31718T, CST), p62 (P0067, Sigma). Primary antibodies were used at a 1:1,000 dilution in Odyssey blocking buffer (LI-COR) and incubated with the blots overnight with shaking at 4°C. Secondary antibodies were IRDye 800 donkey anti-rabbit (LI-COR) and IRDye 680 goat anti-mouse IgG (LI-COR) used at 1:10,000 dilution in Odyssey blocking buffer and incubated with the blots for 45–60 min with shaking at RT. Images were obtained on LI-COR Odyssey IR scanner or Azure Biosystems Blot Imager.

### Kinase panel

Compounds were tested at 3 μM against a panel of 256 human kinases by the Invitrogen SelectScreen Kinase Profiling service. Kinases inhibited by more than 80% relative to controls were considered as hits. Lipid kinases were tested by DiscoverX KINOMEscan Profiling Service in an active site-directed competition binding assay with the compounds of interest in the absence of ATP to predict true thermodynamic interaction affinities. Access to TREEspot software was provided by DiscoverX.

### RNA-Seq

BMDM from three C57BL/6 mice were differentiated as described above and plated in 10% L929-conditioned DMEM medium on day 6 post-differentiation in 6-well Corning Costar flat bottom cell culture plates at a density of 3x10^6^ cells/well and treated with 10 ng/mL of IFNγ. BMDM from three individual mice were plated and treated separately to represent three replica experiments. Next day, BMDM were treated with vehicle (DMSO, 0.1% final concentration), or 2062 at 10 μM for 1, 2, and 6 h in the presence of 10 ng/mL IFNγ (Roche). Cells were washed once with warm PBS and processed for RNA isolation with the Qiagen RNA-easy kit. Quality control of RNA libraries was performed using the BioAnalyzer 2100. Single-end sequencing with 40–50 million reads was performed at the Weill Cornell Genomics Core Facility on Illumina Hi-Seq platform. The RNA-seq data were analyzed by Weill Cornell Genomics Core Facility using DE-seq algorithm. Genes differentially expressed (~2 fold) at FDR 10% and p<0.05 were clustered for further analysis.

### Confocal microscopy

Images were acquired on Zeiss LSM 880 laser scanning confocal microscope with the 63X/1.4 Oil DIC M27 objective. BMDM were seeded in 4- or 8-well Falcon Chambered Cell Culture slides with 0.5-1x10^5^ cells/well in 0.5 mL of 10% LCM in complete DMEM with or without 10 ng/mL IFNγ, allowed to adhere overnight and treated with compounds of interest for the indicated times by replacing the medium with medium containing the indicted final concentrations of compounds. After treatment, BMDM were washed with PBS and fixed with 4% PFA (15 min, RT), washed 3 times (PBS), permeabilized with 0.2% TritonX100/PBS (10 min, RT), washed 3 times with 0.1% TritonX100/PBS, blocked in 0.1% TritonX100/2% BSA/0.1% NaN_3_/PBS (1 h, RT), incubated with primary antibodies at 1:100 dilution in the blocking buffer, incubated overnight at 4 ^o^C with rocking, washed as before, incubated with the secondary antibodies (donkey anti-rabbit Texas-Red (Jackson ImmunoResearch), goat anti-mouse IgG (H+L) Alexa Fluor Plus 488 (Invitrogen) and/or goat anti-rabbit IgG (H+L) Alexa Fluor Plus 647 (Invitrogen) at 1:200 dilution in blocking buffer (1 h, RT) and washed. Nuclei were labeled with Hoechst 33342 Ready Flow Reagent (Invitrogen) during the last wash. The slides were finally rinsed in PBS and mounted with ProLong Antifade mountant (Molecular Probes). Cells were imaged with 405 (UV/Hoechst), 488 (AlexaFluor 488), 594 (Texas Red), or 633 nm (AlexaFluor 647) laser lines. Images were analyzed and microphotographs prepared in ImageJ.

### Lysosomal acidification

We used LysoTracker (Molecular Probes) to label the lysosomal compartment in BMDM according to the manufacturer’s instructions. BMDM were seeded in 8- or 4-well Falcon Chambered Cell Culture slides with 0.5–1.0 × 10^5^ cells/well in 0.5 mL of 10% LCM in complete DMEM with or without 10 ng/mL IFNγ, allowed to adhere overnight and treated with compounds of interest for 24 h by replacing the medium with medium containing the final concentrations of compounds. LysoTracker probe was added next day to the same wells to achieve a final concentration of 50 nM and incubated for 1–2 h at 37 ^o^C. Cells were washed 3 times with warm PBS, immediately fixed in 4% PFA (10 min) and washed 3 times with PBS. Nuclei were labeled with Hoechst 33342 Ready Flow Reagent (Invitrogen) during the last wash. Slides were rinsed in PBS and mounted in ProLong Antifade mountant (Molecular Probes). Microscopic images were taken at 20× magnification and analyzed by ImageJ to calculate LysoTracker fluorescence per cell by measuring total image fluorescence corrected by autofluorescence and divided by the number of nuclei. Over 300 cells were analyzed for each sample. Each experiment was repeated 3 times.

### Cathepsin assay

Magic Red Cathepsin B assay kit (Bio-Rad) was used to determine the cathepsin B-mediated degradative capacity of BMDM exposed to compounds of interest. The experiments were performed as described for the LysoTracker labeling except that the Magic Red Cathepsin B assay kit was used in accordance with the manufacturer’s instructions.

### Intracellular pH measurements

Fluorimetric Intracellular pH Assay Kit (Sigma) was used for intracellular pH measurements in BMDM according to the manufacturer’s instructions. BMDM were plated in 96- and 384-well black clear bottom plates at a density of 1×10^5^ cells/well/100 μL (96-well plates) or 2.5×10^4^ cells/well/25 μl (384-well plates) in 10% LCM in complete DMEM and primed with 10 ng/mL IFNγ. Twenty-four hours later, the medium was replaced with an equal volume of Hank’s Buffer with Hepes (HBSS) plus BCFL-AM dye according to the manufacturer’s protocol. After 30–60 min at 37 ^o^C, test compounds diluted in HBSS were added to the assay plate with 40 μL/well (96-well plates) or 10 μL/well (384-well plates). Ratiometric measurements of fluorescence were recorded on SpectraMax M5 plate reader at 37 ^o^C with λ_ex_ = 505/430 and λ_em_ = 535 nm. pH calibrations were performed in HBSS covering a range of pH (5.6–8.2) in the presence of 50 μM nigericin. Each sample in each experiment was represented by 6 replicate wells.

### Seahorse ECAR measurements

Extracellular acidification rate (ECAR) measurements were performed on a Seahorse XFe96 Analyzer (Agilent) in 96-well plates. RAW 264.7 macrophages were seeded at 1×10^5^ cells/well in 200 μL complete DMEM and treated or not with 10 ng/mL IFNγ and/or 2 μM 2062 (for the wells pre-treated with 2062 for 24 h). No cells were seeded in four corner wells used for background measurements. Compound injection plate was prepared in DMEM at 10x final compound concentration in the test plate. Macrophages were analyzed for 4 h at 37°C with recordings every 5 min. Compounds were injected in 20 μL 18 min after the start of the run. Final DMSO content after compound addition to the wells was 0.05%.

### Ca^2+^ measurements

RAW 264.7 or BMDM macrophages were used as indicated in 384-well plates in complete DMEM (RAW 264.7 cells) or DMEM plus 10% LCM (BMDM) with 25 μL/well to achieve 30,000–40,000 cells/well and treated overnight with or without 10 ng/mL IFNγ and/or 3 μM 2062. HBSS without cells was plated in the perimeter wells (Columns 1 and 24; Rows A and P). Fura2 AM was added to wells with cells in 25 μL medium with 10 μL of HBSS with 20 mM HEPES, pH 7.3 (HBSS/HEPES) to give a final loading concentration of 2.5 μM Fura2 AM. The Fura2 AM stock was prepared at 1 mM in DMSO (50 μL) and premixed for 10 min with an equal volume of Pluronic F-127 (50 μL) (20% solution in DMSO, ThermoFisher) to facilitate dispersion and uptake of the dye. This mix (90 μM) was then added to 5 mL of HBSS/HEPES before addition to the wells. After 1.5 h at 37 ^o^C, 30 μL of the medium was removed and replaced with 25 μL of HBSS/HEPES with or without Ca^2+^, 3 μM 2062 and/or 10 ng/mL IFNγ. Plates were incubated for another hour at RT or 37 ^o^C to complete Fura2 AM de-esterification inside the cells and equilibrate the cells to the temperature at which the experiment was performed (RT or 37°C). Ratiometric fluorescence Fura2 measurements were recorded on a Hamamatsu FDSS-60000 fluorimeter every 4 sec. Test compounds (2062, ionomycin) in HBSS/HEPES were added at 10 and 30 min after the start of measurements in a volume of 10 μL/well. Each sample was represented by 7–14 replicate wells and the measurements were averaged to obtain the traces presented in the figures.

### CRISPR/Cas9 deletion of TFEB

RAW 264.7 macrophages were cultured in DMEM supplemented with 10% FBS, 1% pyruvate, 1% HEPES and 1% L-glutamine and subcultured as they reached confluency. Guide RNAs (gRNA) for TFEB were designed with the help of the online CRISPR Design Tool (http://crispr.mit.edu). The following gRNA’s were cloned into the gRNA scaffold of the pX458-SpCas9 plasmid: 5’-CATGCAGCTCATGCGGGAGC-3’ (1), 5’-CATCACTGTCTGGGCTCAT-3’ (2). The plasmid was sequenced, maxi-prepped and used to transfect RAW 264.7 macrophages in a 12-well plate with 0.5 μg of plasmid/well and 2 μL jetPRIME transfection reagent (Polyplus-transfection SA). 48–72 h after transfection, cells were collected with 0.25% trypsin/EDTA. GFP-positive cells were collected by FACS and cultured in a 24-well plate till confluency. The effectiveness of gRNA was confirmed by T7 endonuclease I-cutting assay. Clonal cell populations were subcultured. Their TFEB genomic region was PCR-amplified and submitted for sequencing. Knockout clones and wild-type clones that had gone through the same procedures were identified from the sequencing data and cryopreserved.

## Supporting information

S1 Fig 2062 potentiates the effects of low dose INH in Mtb-infected BMDM, is not cytotoxic and has no effect on growth of Mtb in axenic culture. **(A)** BMDM exposed or not to IFNγ (10 ng/mL) were infected with Mtb H37Rv at MOI of 0.1 for 4 hours, washed, and left untreated (solid black lines) or treated with 2062 alone (solid red lines), INH alone (dashed black lines) or the combination of 2062 and INH (dashed red lines). 2062 was used at 3 μM and INH at 0.1 μg/ml. **(B)** MTS assay for viability of human cell lines (HeLa, MCS-7) and mouse BMDM in the presence of increasing concentrations of 2062. Cells were plated at 5 x 10^5^ cell/well in a 96 well plate and exposed to compound for 48 h. **(C)** Mtb H37Rv was incubated in the presence of 2-fold serial dilutions of 2062, rifampin or serial dilutions of rifampin in the presence of 10 or 20 μM 2062. Optical density was determined after 7 d incubation at 37°C in 5% CO_2_, 95% humidified air. Data are expressed as percent growth relative to DMSO containing wells.
(TIF)

S2 Fig Hemogram of uninfected C57BL/6 mice treated IP with 50 mg/kg 2062 daily for 7 days. (TIF)

S3 Fig The complete blood chemistry panel (CBC) of uninfected C57BL/6 mice treated IP with 50 mg/kg 2062 daily for 7 days. (TIF)

S4 Fig Pharmacokinetic studies of 2062. Blood levels of 2062 in mice after (A) IP dose of 50 mg/kg in 5%NMP/45%PEG400/50%D5W (A, red) or PO dose of 50 mg/kg in 0.5%CMC/0.25%Tween80 (A, black) and (B) multiple PO doses at 30 mg/kg (black) or 50 mg/kg (red) in 0.5%CMC/0.25%Tween80.
(TIF)

S5 Fig Mouse Mtb infection and IP treatment with 2062 and mouse weight during the course of treatment with 2062 and/or rifampin in exp. **4**. Mice were infected with Mtb by inhalation and disease was allowed to develop for 2 weeks. **(A)** Treatment by IP administration daily for 6 weeks beginning on day 15, followed by plating of lungs (closed symbols) and spleens (open symbols) for CFU. Mice received vehicle alone (black symbols) or 2062 (50 mg/kg; red symbols) in two independent experiments (exp. 1, circles; exp. 2, squares). The asterisk indicates that no CFUs were recovered in spleens from 2 mice. **(B)** All mice in exp. 4 were weighed daily during PO treatment with vehicle (black), 30 mg/kg 2062 (red), 50 mg/kg 2062 (blue), 3 mg/kg rifampin (black dashed), 30 mg/kg 2062 + 3 mg/kg rifampin (red dashed) and 50 mg/kg 2062 + 3 mg/kg rifampin (blue dashed). **(C)** Structure of the 2062 probe compound.
(TIF)

S6 Fig Analysis of the proteomics data from the pulldown experiments with the 2062 probe. Hits were selected based on intensity of the MS data from the triplicate pulldown samples with the active 2062 probe as compared with the triplicate samples from the inactive probe. Significantly different hits are labeled with a plus sign.
(TIF)

S7 Fig Profile analysis of 2062 against 256 human kinases. 2062 was tested at 3 μM and remaining activity relative to control is provided for each of the 256 human kinases.
(TIF)

S8 Fig Impact of 2062 on macrophage intracellular pH and Ca^2+^ stores. **(A)** 2062 and IFNγ each alkalinize the cytosol of BMDM as measured by BCFL fluorescence. Each data point represents the means ± SD of 6 replica wells. Shown is a representative of two independent experiments. **(B)** Addition of 2062 (5 μM) quickly increases RAW ECAR with or without pretreatment overnight with IFNγ (10 ng/mL) and/or 2062 (2 μM). Each data point represents the means ± SD of 4 replica wells. **(C)** Impact of 2062 on intracellular Ca^2+^ levels and Ca^2+^ fluxes induced by ionomycin (Ion, 10 μM) in Ca^2+^-replete media in IFNγ-activated (+) or naïve (-) RAW 264.7 macrophages with or without overnight pretreatment with 2 μM 2062 (2062PT), as measured with Fura 2. Arrows depict ionomycin additions. **(D)** As in **(C)** but in a Ca^2+^-depleted medium. Each data point is represented by 7–14 replicate wells; measurements were averaged to obtain the traces presented in the figures. Individual traces are displaced along the X axis for clear peak visualization; time 0 for each trace corresponds to its beginning.
(TIF)

S9 Fig Confocal microscopy of TFE3 cellular localization. BMDM were primed with 10 ng/mL IFNγ or not for 24 h and treated or not with 5 μM 2062 for 1 h, cells were fixed and stained with anti-TFE3 antibody (Sigma, HPA023881; 1:100) and Hoechst 33342 as described in the Methods. Shown are BMDM not treated with 2062 primed with 10 ng/mL IFNγ (bottom) or not (top). TFE3 is color-coded red, Hoechst 33342 is blue.
(TIF)

S10 Fig RNA-Seq analysis of the CLEAR genes regulated by 2062 at 1 and 2 h post treatment. (TIF)

S11 Fig Western Blot analysis of p62, cathepsin B and actin in Mtb-infected BMDM treated with 2062, rifampin or their combination. **(A)** ImageJ analysis of p62, cathepsin B and actin bands from Western Blots performed on cytoplasmic extracts of Mtb-infected BMDM 3 days (from Fig 6G) post treatment with 2062 (1 μM), rifampin (0.5 μM) or their combination. **(B)** ImageJ analysis as in (A) but on independent samples 6 days post treatment (from S11C Fig). **(C)** Western Blot of cytoplasmic extracts of Mtb-infected BMDM after 6 days of treatment with 2062 (1 μM), rifampin (0.5 μM) or their combination.
(TIF)

S12 Fig 2062 inhibits ERK1/2 and mTORC1 and activates TFEB in a Ca^2+^-dependent manner. 2062 inhibits mTORC1 and ERK1/2 kinases in a time- **(A)** and dose-dependent manner **(B)**. IFNγ-activated (10 ng/mL) BMDM were treated with 5 μM 2062 for the indicated times or at the indicated concentrations for 1 h. Soluble extracts separated on 10% SDS-PAGE were probed with antibodies to p-S6K, S6K, p-ERK1/2 and ERK1/2. **(C)** Chelation of free intracellular Ca^2+^ with BAPTA-AM prevents TFEB nuclear translocation. BMDM were pre-treated with increasing concentrations with BAPTA-AM (4, 10, 25 μM) for 1 h and then exposed to 3 μM 2062 for 2 h. Shown are nuclear extracts probed with anti-TFEB, anti-NFAT and corresponding cytosolic extracts probed with anti-actin. **(D)** 2062 promotes TFEB nuclear translocation in a dose-dependent manner in the presence of extracellular Ca^2+^ but not in Ca^2+^-free medium.
(TIF)

S13 Fig Mtb infection of WT and TFEB-deleted RAW macrophages and treatment with 2062, rifampin, or their combination. RAW macrophages (WT or TFEB KO) exposed (+, open symbols) or not (-, solid symbols) to IFNγ (10 ng/mL) were infected with Mtb H37Rv at MOI of 1 for 4 hours, washed, and left untreated (solid black lines) or treated with 2062 alone (solid red lines), rifampin alone (solid blue lines) or the combination of 2062 and rifampin (solid purple lines). 2062 was used at 1 μM and rifampin at 0.25 μM. Cells were lysed at the indicated times for determination of CFU. Results are mean ± SD of triplicate wells in a single experiment. Two independent experiments are shown. P values were calculated by unpaired t-test; ns, not significant.
(TIF)

S14 Fig The effect of TFEB activators on Mtb growth in culture and in Mtb-infected BMDM. Mtb mc^2^6220 Δ*panCD*Δ*lysA* **(A, left)** or H37Rv **(B, right)** was incubated in the presence of 2-fold serial dilutions of Torin 1 **(A, left)** or digoxin (DG), alexidine (AD) and ikarugamycin (IKA) **(A, right)** and optical density was determined after 7d incubation at 37°C in 5% CO_2_, 95% humidified air. Data are expressed as percent growth relative to DMSO containing wells. **(B)** Nuclear extracts from non-treated (NT) or Torin 1 (Tor1, 1 μM) (left panel) and DG (300 nM), AD (3 μM), and IKA (1 μM) (right panel) treated BMDM separated on SDS-PAGE, transferred to nitrocellulose and probed with anti-TFEB antibody (1:1000). **(C)** The effects of DG (left) or AD (right) on Mtb viability in infected BMDM. BMDM exposed or not to IFNγ (10 ng/mL) were infected with Mtb H37Rv at MOI of 0.1 for 4 hours, washed, and left untreated (solid black lines) or treated with 300 nM DG alone (left, solid red lines), or 3 μM AD alone (right, solid red lines), rifampin alone (dashed black lines) or the combination of DG (left) or AD (right) and rifampin (dashed red lines). Rifampin at 0.5 μM. Cells were lysed at the indicated times for determination of CFU. Results are mean ± SD of triplicate wells in a single experiment representative of 2 independent experiments. **(D)** The effects of ikarugamycin on Mtb viability in infected BMDM. Experiment was carried out as described in panel C except IKA was used at 300 nM.
(TIF)

S15 Fig Structures and ^1^H NMR features of the active (2062) and inactive (2833) analogs. (TIF)
