# Supplementary figures and images for: Potentiation of rifampin activity in a mouse model of tuberculosis by activation of host transcription factor EB
Source: PLoS Pathog. 2020 Jun 23;16(6):e1008567. doi: 10.1371/journal.ppat.1008567 (PMC7337396; doi:10.1371/journal.ppat.1008567)

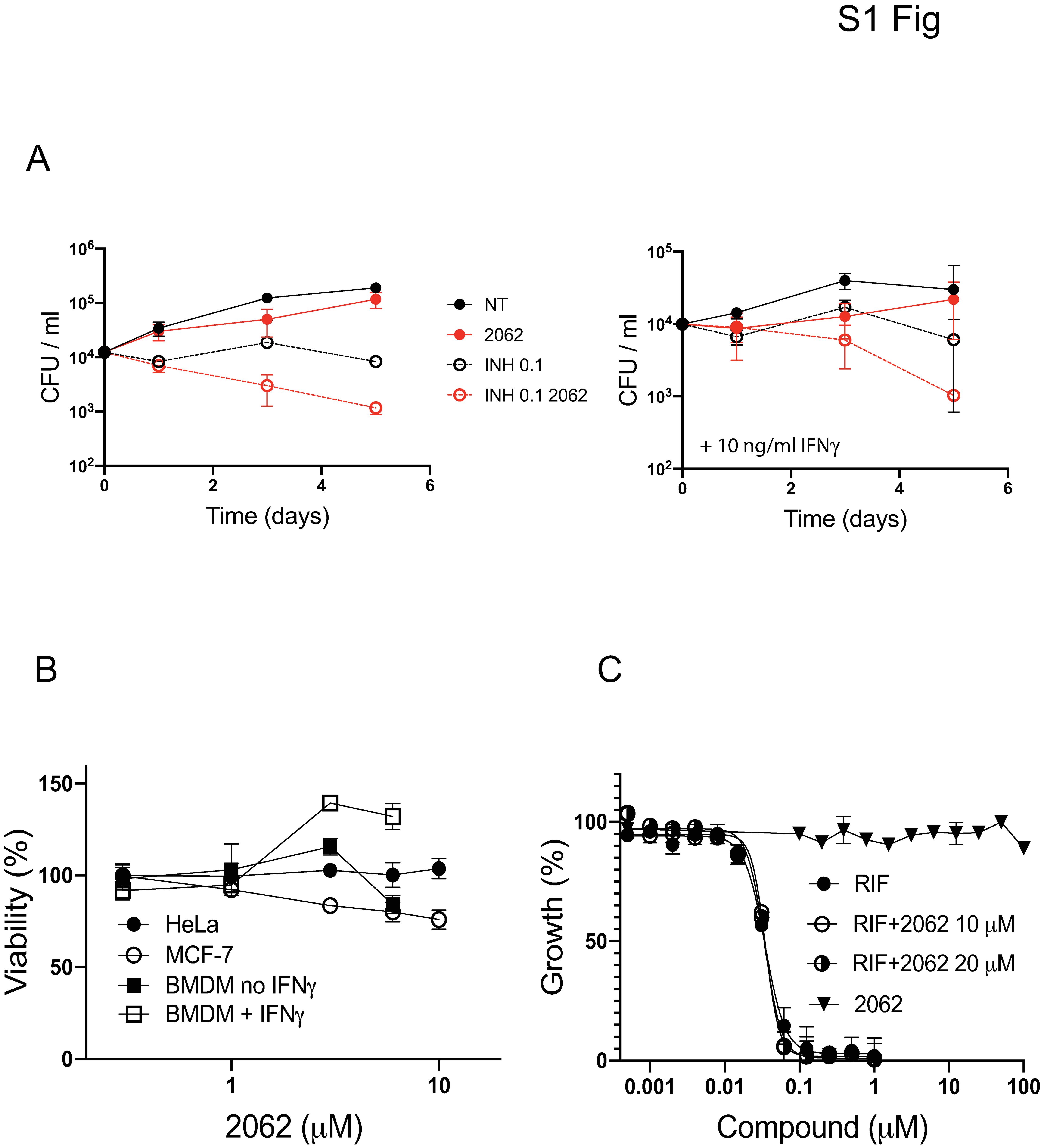

Supplement: S1 Fig — (A) BMDM exposed or not to IFNγ (10 ng/mL) were infected with Mtb H37Rv at MOI of 0.1 for 4 hours, washed, and left untreated (solid black lines) or treated with 2062 alone (solid red lines), INH alone (dashed black lines) or the combination of 2062 and INH (dashed red lines). 2062 was used at 3 μM and INH at 0.1 μg/ml. (B) MTS assay for viability of human cell lines (HeLa, MCS-7) and mouse BMDM in the presence of increasing concentrations of 2062. Cells were plated at 5 x 10^5^ cell/well in a 96 well plate and exposed to compound for 48 h. (C) Mtb H37Rv was incubated in the presence of 2-fold serial dilutions of 2062, rifampin or serial dilutions of rifampin in the presence of 10 or 20 μM 2062. Optical density was determined after 7 d incubation at 37°C in 5% CO2, 95% humidified air. Data are expressed as percent growth relative to DMSO containing wells. (TIF) [file ppat.1008567.s001.tif]

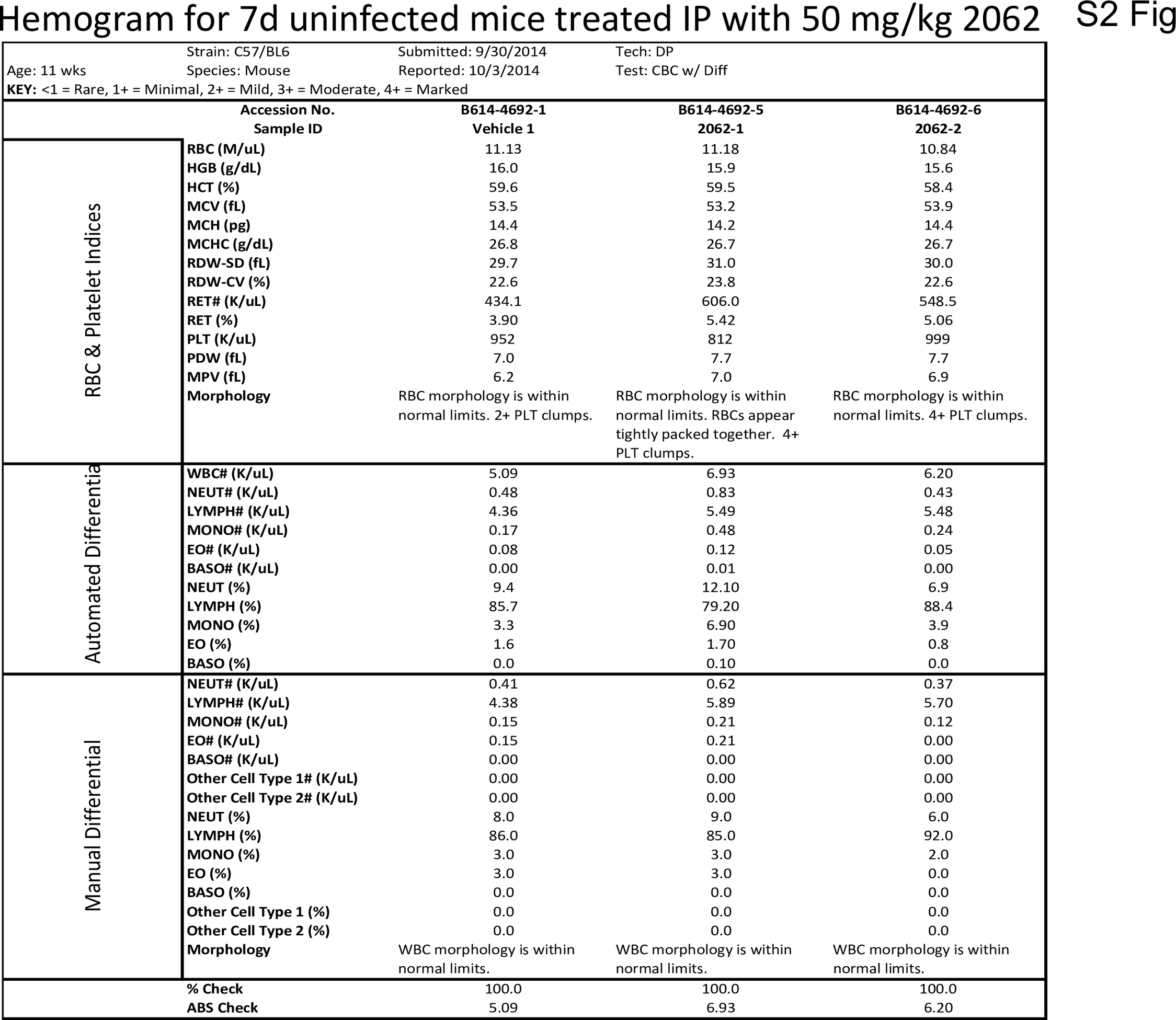

Supplement: S2 Fig — (TIF) [file ppat.1008567.s002.tif]

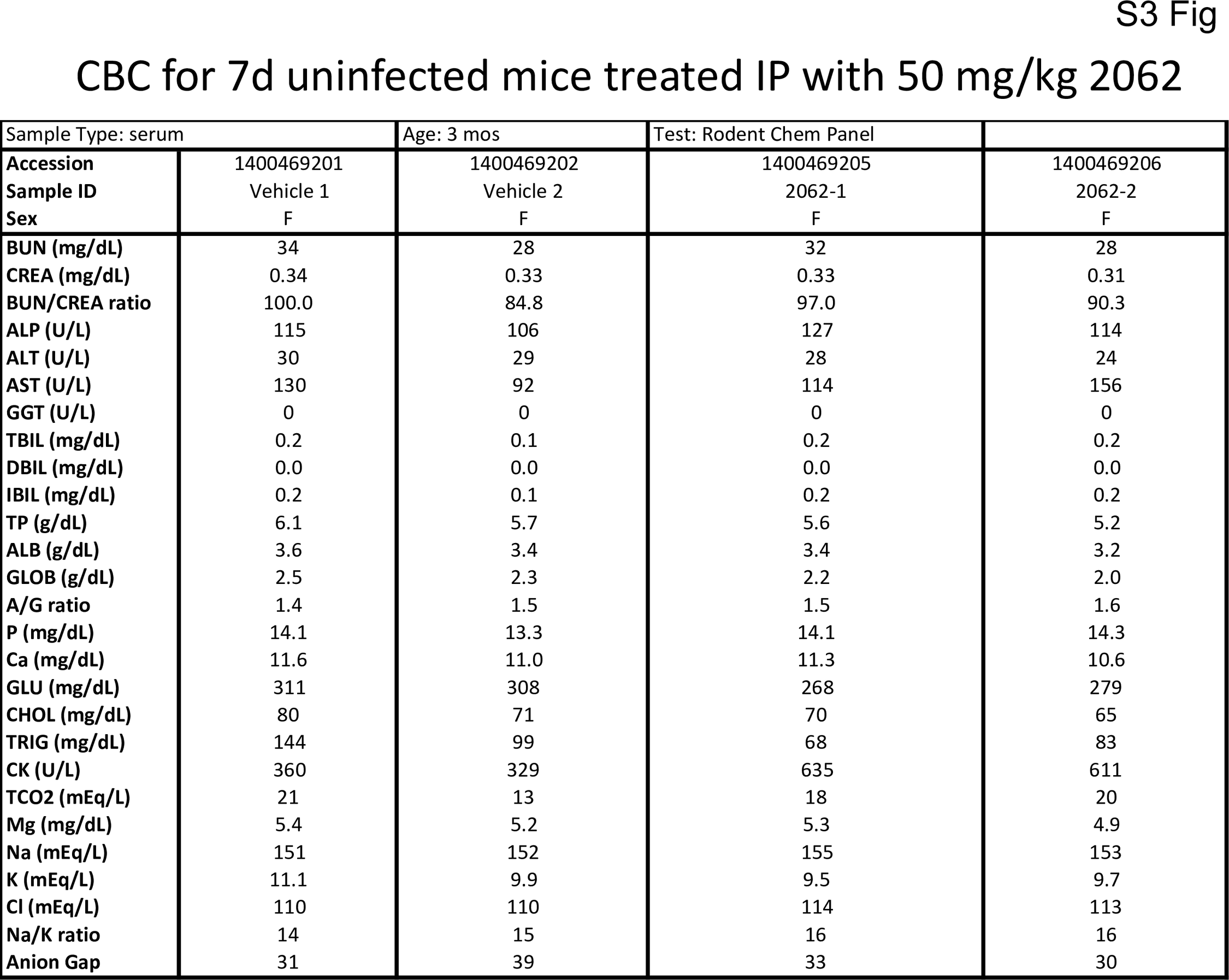

Supplement: S3 Fig — (TIF) [file ppat.1008567.s003.tif]

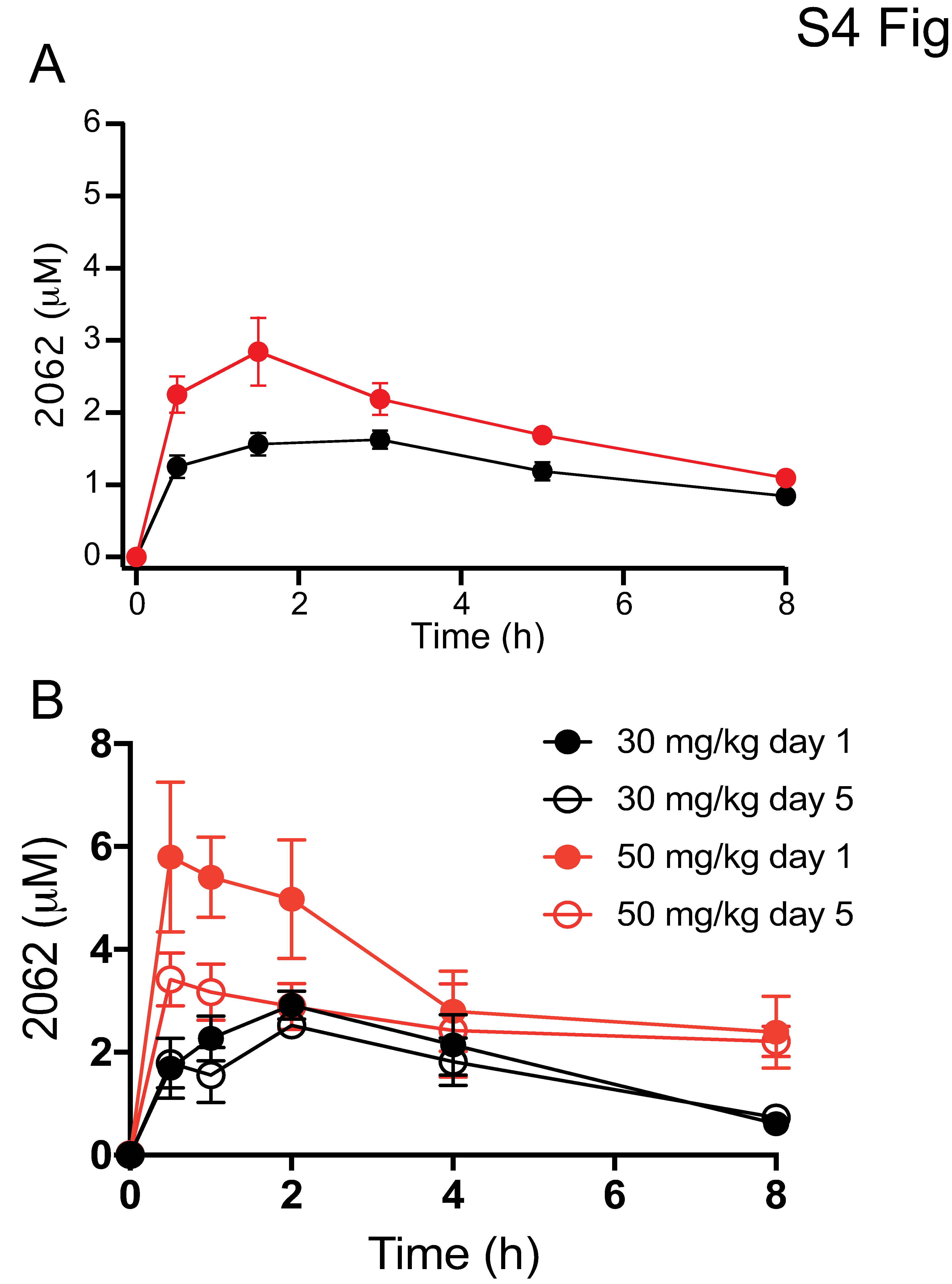

Supplement: S4 Fig — Blood levels of 2062 in mice after (A) IP dose of 50 mg/kg in 5%NMP/45%PEG400/50%D5W (A, red) or PO dose of 50 mg/kg in 0.5%CMC/0.25%Tween80 (A, black) and (B) multiple PO doses at 30 mg/kg (black) or 50 mg/kg (red) in 0.5%CMC/0.25%Tween80. (TIF) [file ppat.1008567.s004.tif]

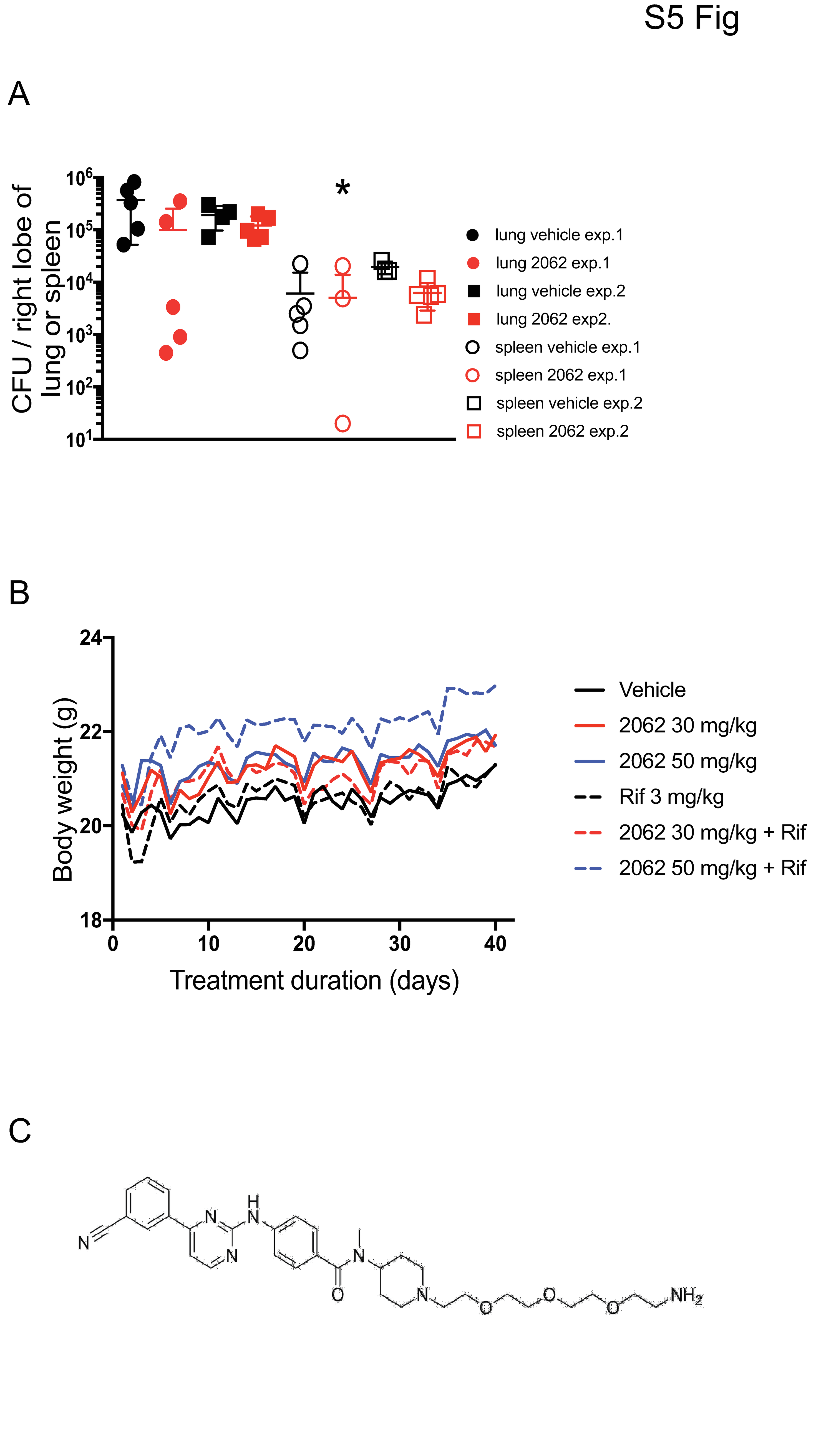

Supplement: S5 Fig — 4. Mice were infected with Mtb by inhalation and disease was allowed to develop for 2 weeks. (A) Treatment by IP administration daily for 6 weeks beginning on day 15, followed by plating of lungs (closed symbols) and spleens (open symbols) for CFU. Mice received vehicle alone (black symbols) or 2062 (50 mg/kg; red symbols) in two independent experiments (exp. 1, circles; exp. 2, squares). The asterisk indicates that no CFUs were recovered in spleens from 2 mice. (B) All mice in exp. 4 were weighed daily during PO treatment with vehicle (black), 30 mg/kg 2062 (red), 50 mg/kg 2062 (blue), 3 mg/kg rifampin (black dashed), 30 mg/kg 2062 + 3 mg/kg rifampin (red dashed) and 50 mg/kg 2062 + 3 mg/kg rifampin (blue dashed). (C) Structure of the 2062 probe compound. (TIF) [file ppat.1008567.s005.tif]

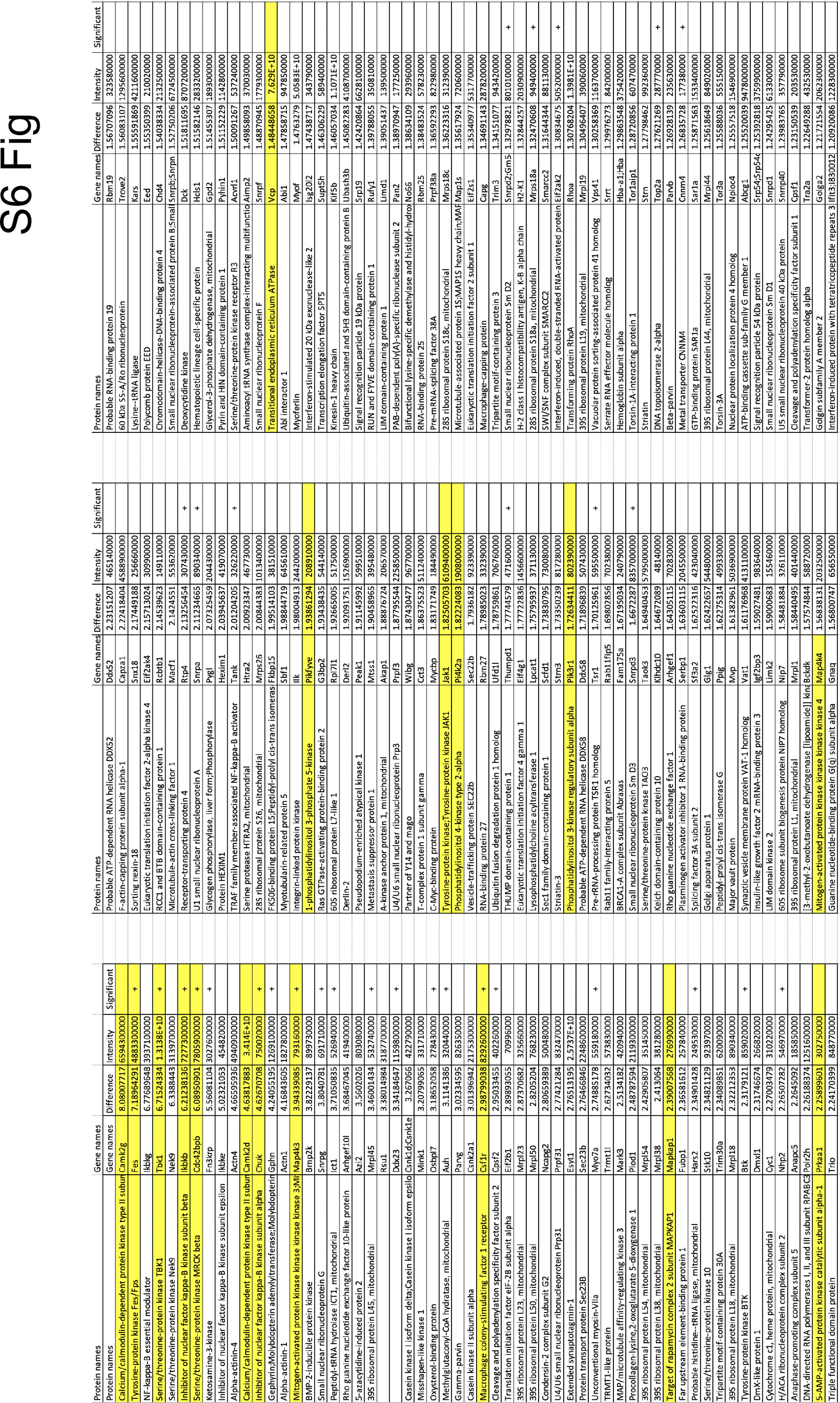

Supplement: S6 Fig — Hits were selected based on intensity of the MS data from the triplicate pulldown samples with the active 2062 probe as compared with the triplicate samples from the inactive probe. Significantly different hits are labeled with a plus sign. (TIF) [file ppat.1008567.s006.tif]

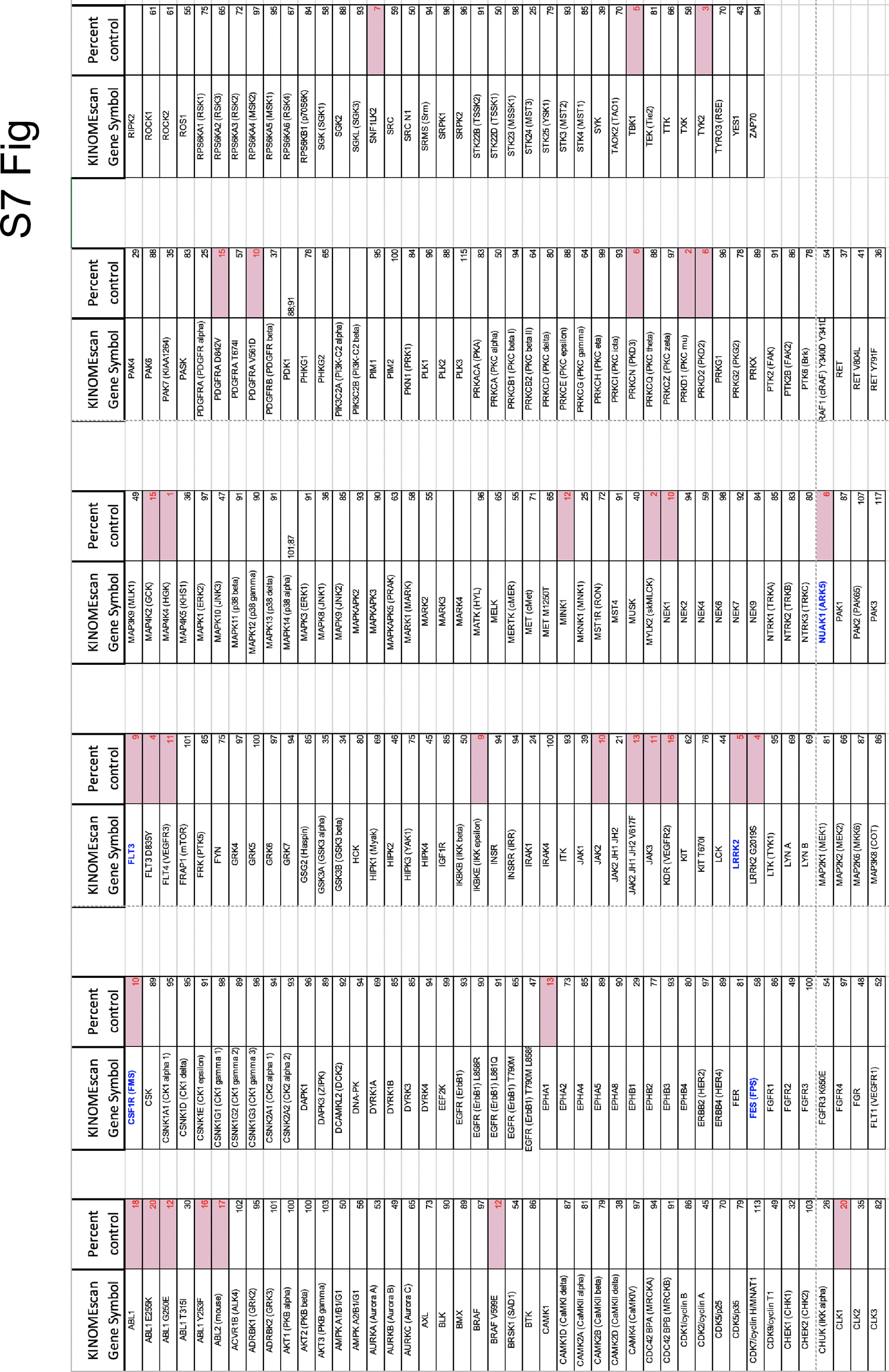

Supplement: S7 Fig — 2062 was tested at 3 μM and remaining activity relative to control is provided for each of the 256 human kinases. (TIF) [file ppat.1008567.s007.tif]

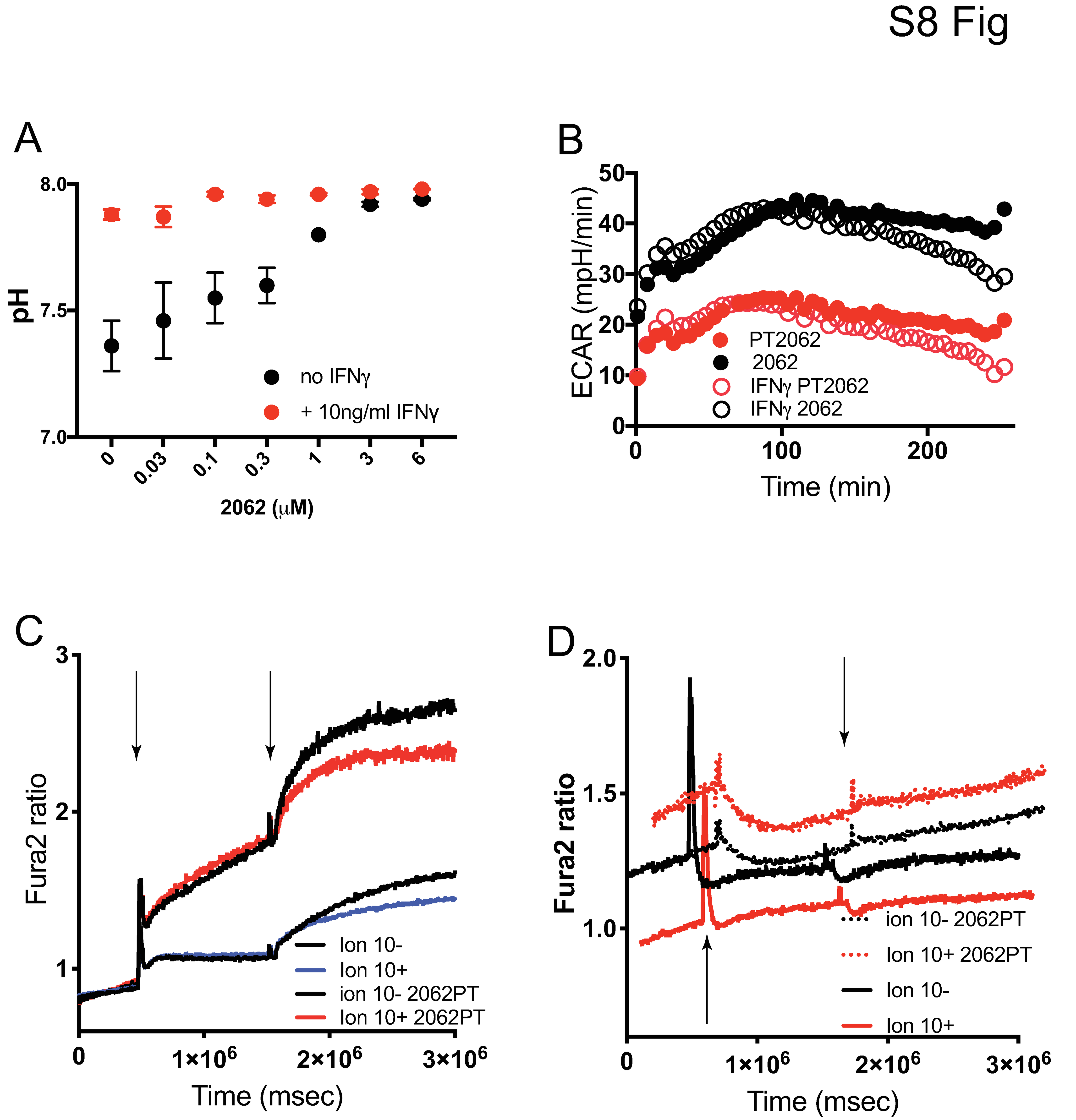

Supplement: S8 Fig — (A) 2062 and IFNγ each alkalinize the cytosol of BMDM as measured by BCFL fluorescence. Each data point represents the means ± SD of 6 replica wells. Shown is a representative of two independent experiments. (B) Addition of 2062 (5 μM) quickly increases RAW ECAR with or without pretreatment overnight with IFNγ (10 ng/mL) and/or 2062 (2 μM). Each data point represents the means ± SD of 4 replica wells. (C) Impact of 2062 on intracellular Ca2+ levels and Ca2+ fluxes induced by ionomycin (Ion, 10 μM) in Ca2+-replete media in IFNγ-activated (+) or naïve (-) RAW 264.7 macrophages with or without overnight pretreatment with 2 μM 2062 (2062PT), as measured with Fura 2. Arrows depict ionomycin additions. (D) As in (C) but in a Ca2+-depleted medium. Each data point is represented by 7–14 replicate wells; measurements were averaged to obtain the traces presented in the figures. Individual traces are displaced along the X axis for clear peak visualization; time 0 for each trace corresponds to its beginning. (TIF) [file ppat.1008567.s008.tif]

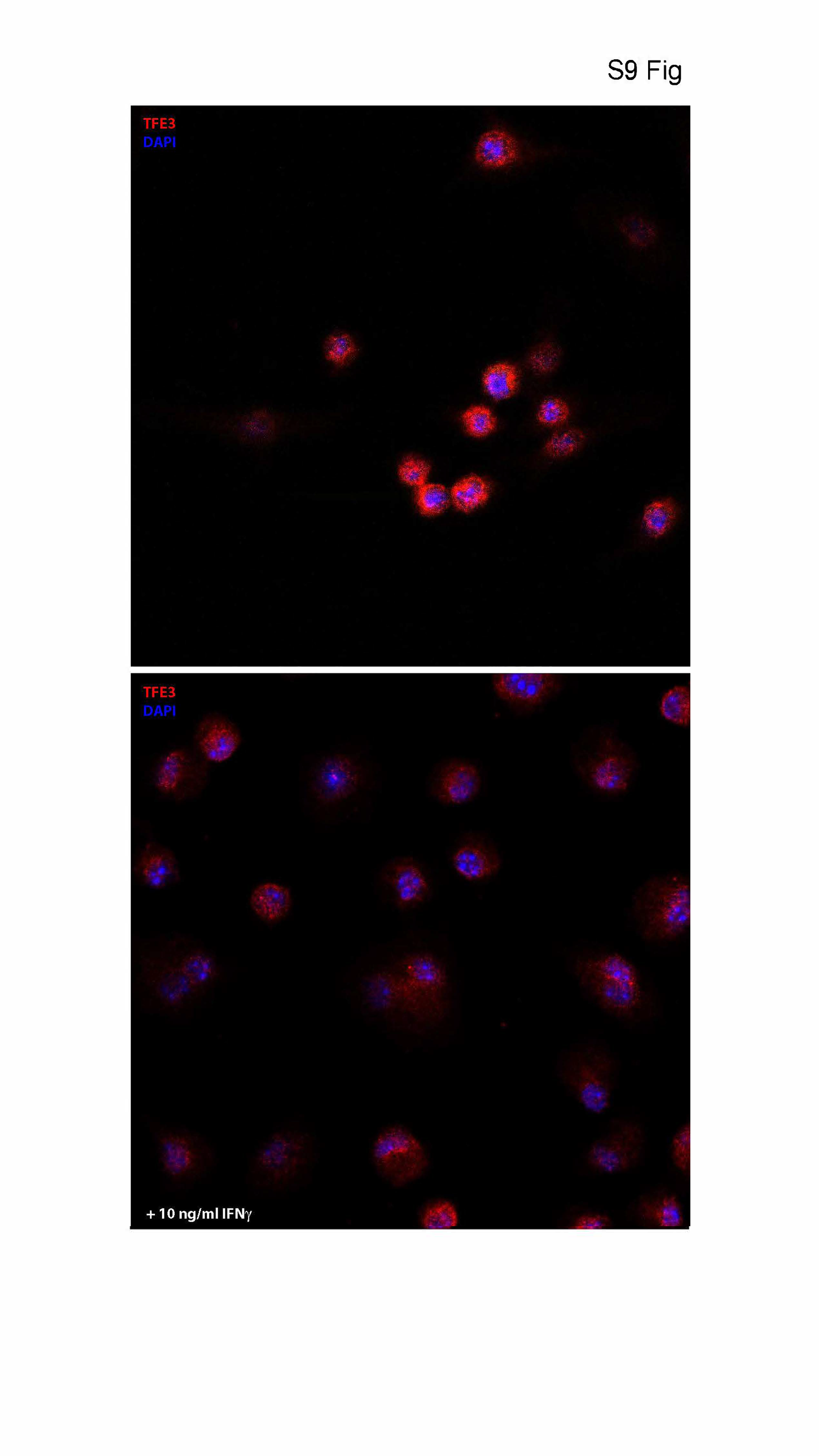

Supplement: S9 Fig — BMDM were primed with 10 ng/mL IFNγ or not for 24 h and treated or not with 5 μM 2062 for 1 h, cells were fixed and stained with anti-TFE3 antibody (Sigma, HPA023881; 1:100) and Hoechst 33342 as described in the Methods. Shown are BMDM not treated with 2062 primed with 10 ng/mL IFNγ (bottom) or not (top). TFE3 is color-coded red, Hoechst 33342 is blue. (TIF) [file ppat.1008567.s009.tif]

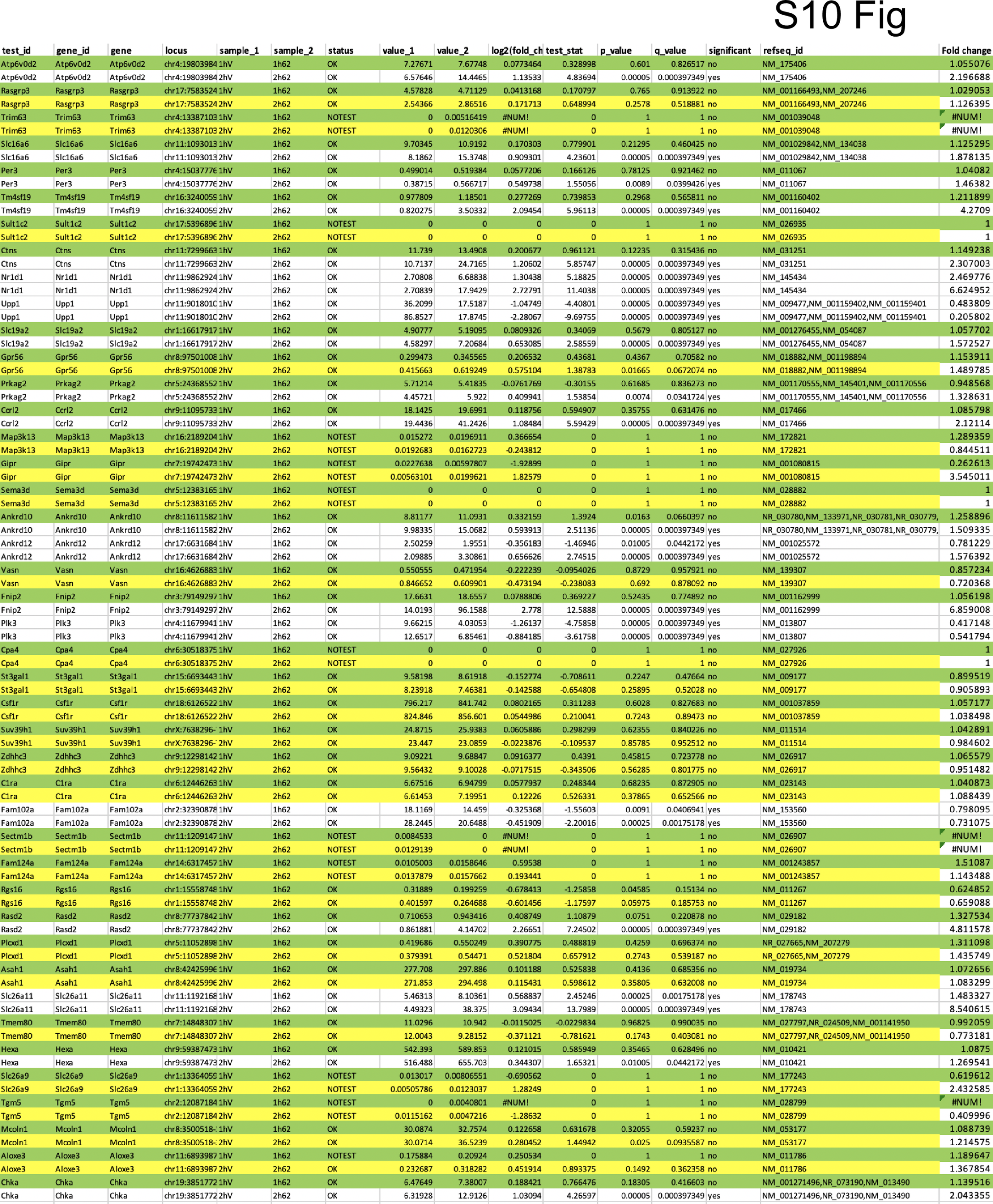

Supplement: S10 Fig — (TIF) [file ppat.1008567.s010.tif]

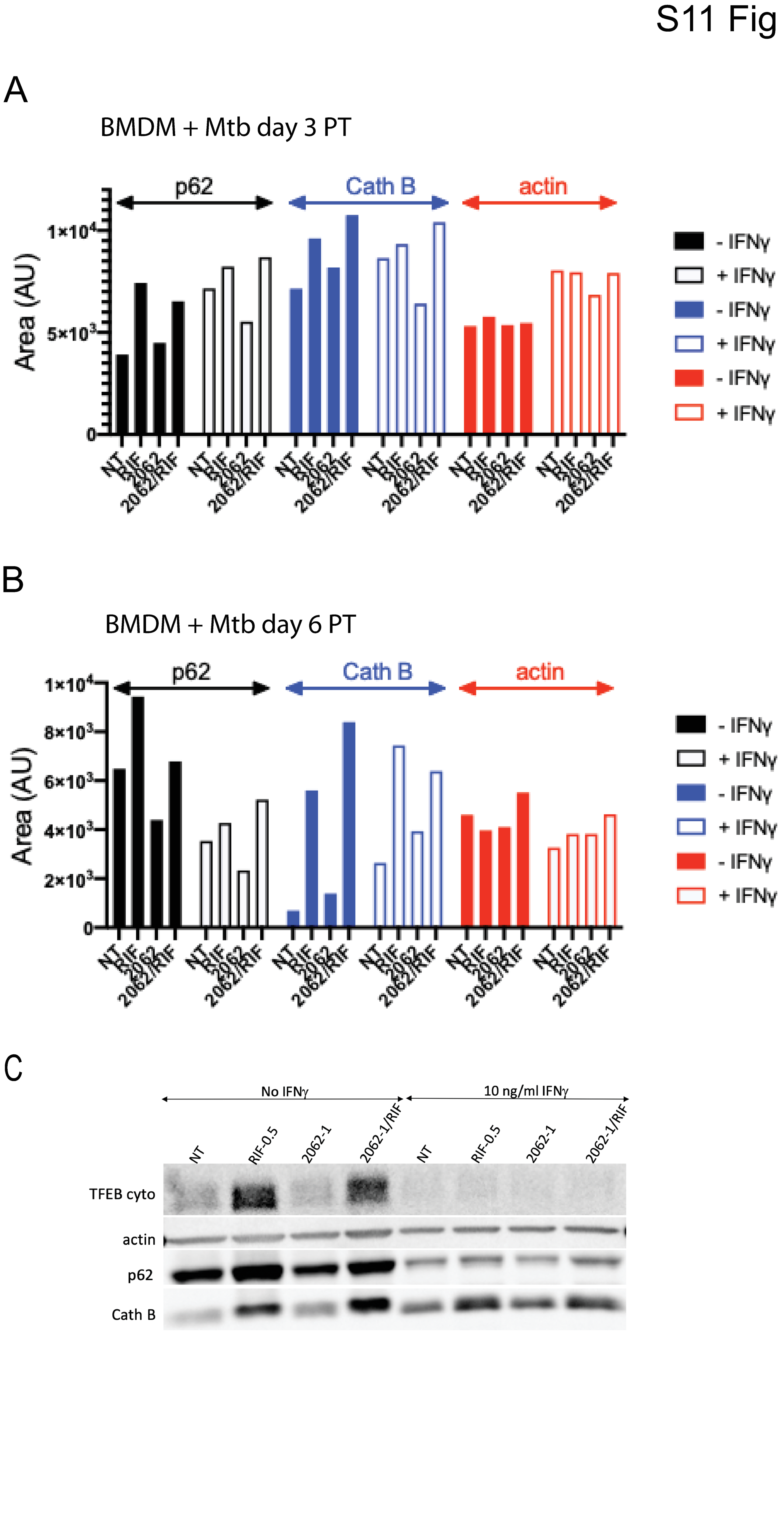

Supplement: S11 Fig — (A) ImageJ analysis of p62, cathepsin B and actin bands from Western Blots performed on cytoplasmic extracts of Mtb-infected BMDM 3 days (from Fig 6G) post treatment with 2062 (1 μM), rifampin (0.5 μM) or their combination. (B) ImageJ analysis as in (A) but on independent samples 6 days post treatment (from S11C Fig). (C) Western Blot of cytoplasmic extracts of Mtb-infected BMDM after 6 days of treatment with 2062 (1 μM), rifampin (0.5 μM) or their combination. (TIF) [file ppat.1008567.s011.tif]

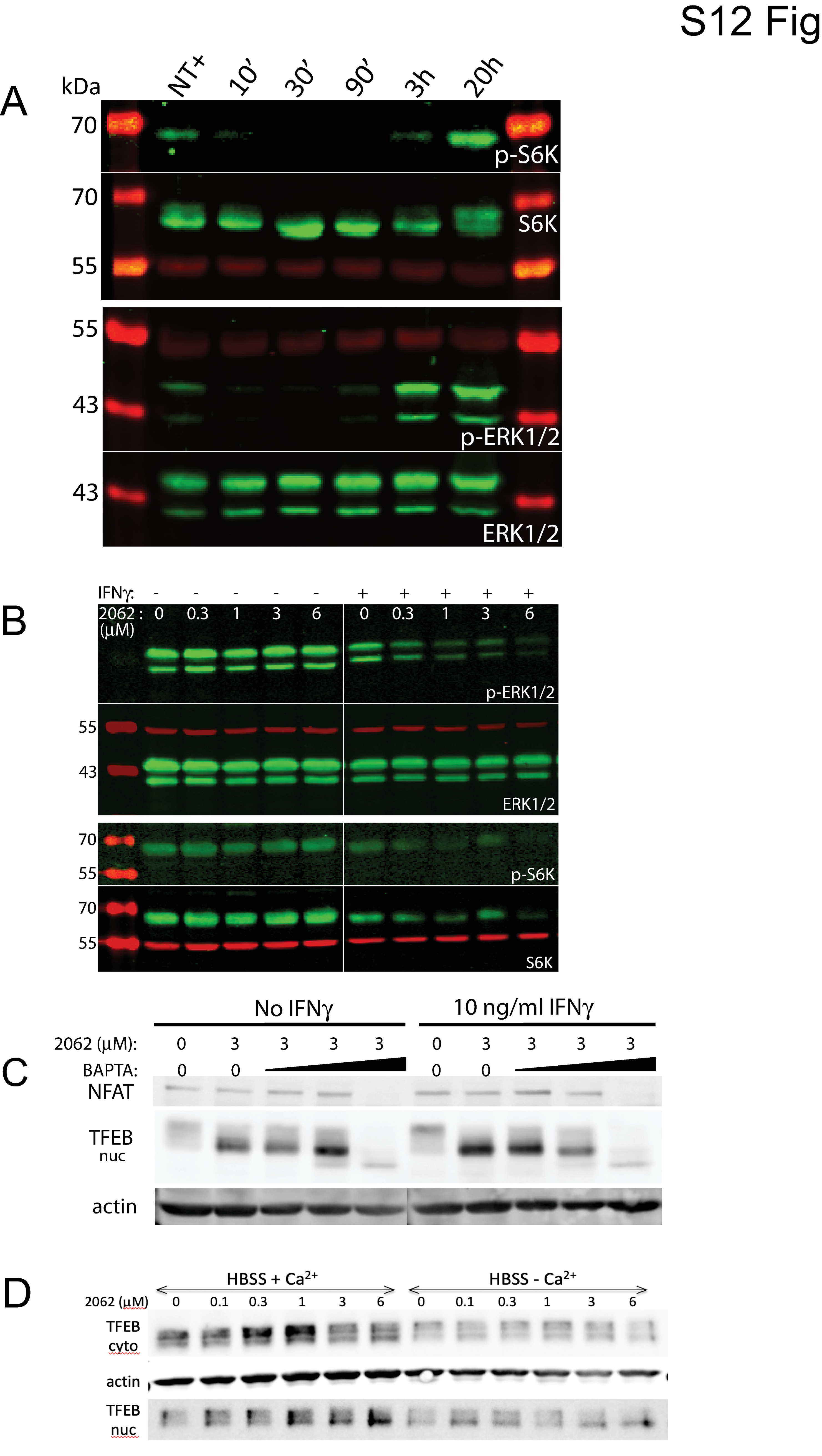

Supplement: S12 Fig — 2062 inhibits mTORC1 and ERK1/2 kinases in a time- (A) and dose-dependent manner (B). IFNγ-activated (10 ng/mL) BMDM were treated with 5 μM 2062 for the indicated times or at the indicated concentrations for 1 h. Soluble extracts separated on 10% SDS-PAGE were probed with antibodies to p-S6K, S6K, p-ERK1/2 and ERK1/2. (C) Chelation of free intracellular Ca2+ with BAPTA-AM prevents TFEB nuclear translocation. BMDM were pre-treated with increasing concentrations with BAPTA-AM (4, 10, 25 μM) for 1 h and then exposed to 3 μM 2062 for 2 h. Shown are nuclear extracts probed with anti-TFEB, anti-NFAT and corresponding cytosolic extracts probed with anti-actin. (D) 2062 promotes TFEB nuclear translocation in a dose-dependent manner in the presence of extracellular Ca2+ but not in Ca2+-free medium. (TIF) [file ppat.1008567.s012.tif]

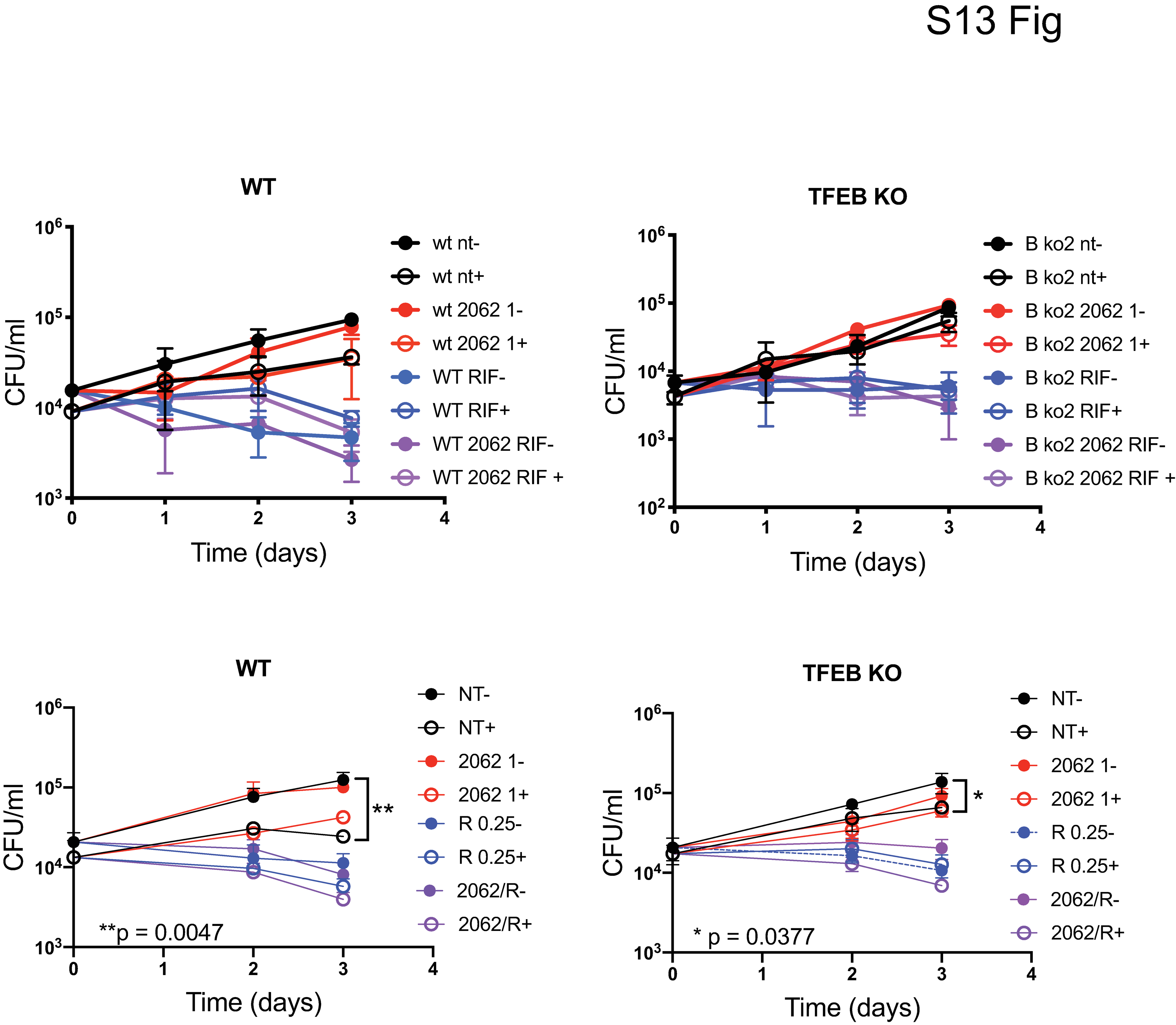

Supplement: S13 Fig — RAW macrophages (WT or TFEB KO) exposed (+, open symbols) or not (-, solid symbols) to IFNγ (10 ng/mL) were infected with Mtb H37Rv at MOI of 1 for 4 hours, washed, and left untreated (solid black lines) or treated with 2062 alone (solid red lines), rifampin alone (solid blue lines) or the combination of 2062 and rifampin (solid purple lines). 2062 was used at 1 μM and rifampin at 0.25 μM. Cells were lysed at the indicated times for determination of CFU. Results are mean ± SD of triplicate wells in a single experiment. Two independent experiments are shown. P values were calculated by unpaired t-test; ns, not significant. (TIF) [file ppat.1008567.s013.tif]

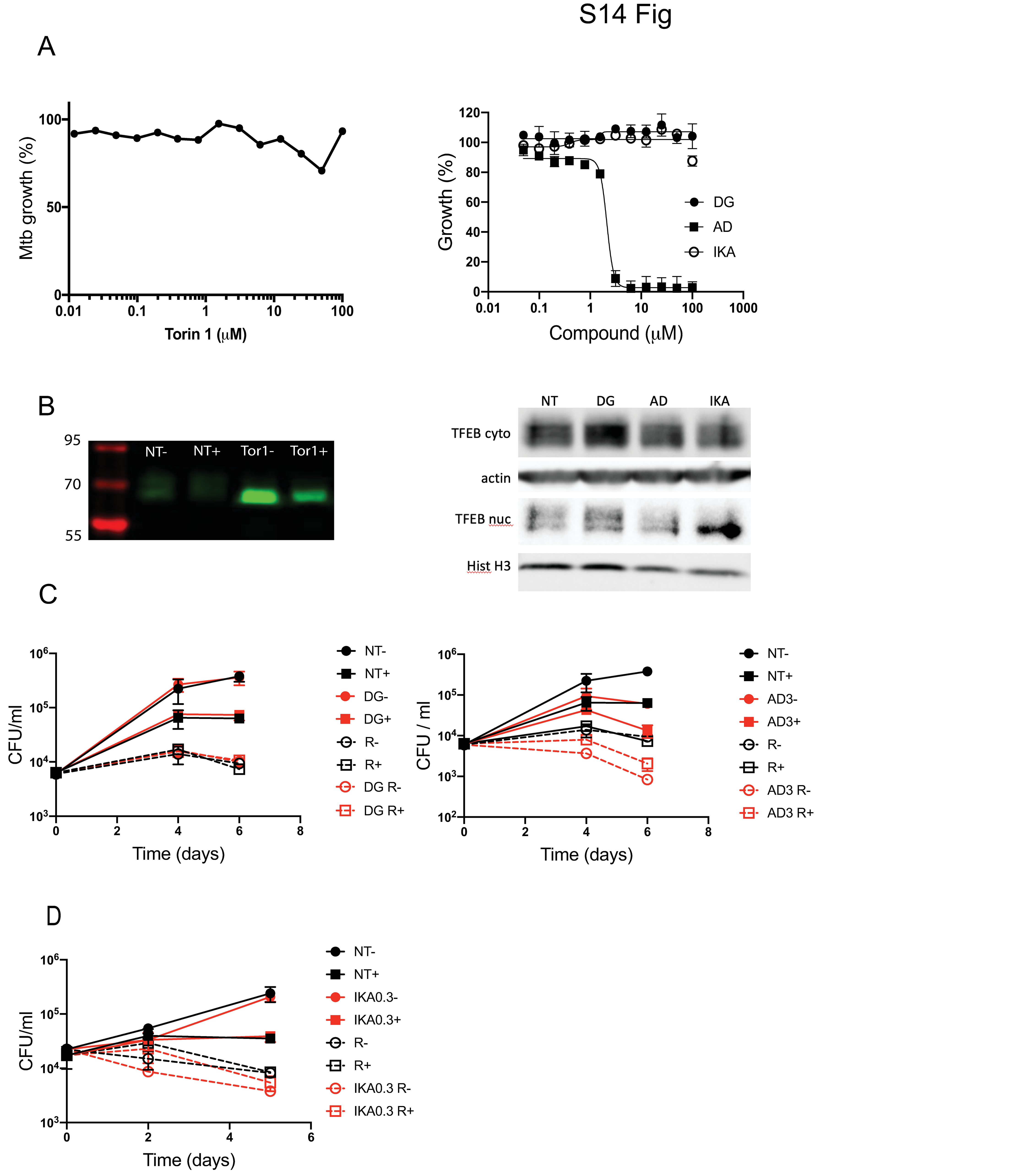

Supplement: S14 Fig — Mtb mc26220 ΔpanCDΔlysA (A, left) or H37Rv (B, right) was incubated in the presence of 2-fold serial dilutions of Torin 1 (A, left) or digoxin (DG), alexidine (AD) and ikarugamycin (IKA) (A, right) and optical density was determined after 7d incubation at 37°C in 5% CO2, 95% humidified air. Data are expressed as percent growth relative to DMSO containing wells. (B) Nuclear extracts from non-treated (NT) or Torin 1 (Tor1, 1 μM) (left panel) and DG (300 nM), AD (3 μM), and IKA (1 μM) (right panel) treated BMDM separated on SDS-PAGE, transferred to nitrocellulose and probed with anti-TFEB antibody (1:1000). (C) The effects of DG (left) or AD (right) on Mtb viability in infected BMDM. BMDM exposed or not to IFNγ (10 ng/mL) were infected with Mtb H37Rv at MOI of 0.1 for 4 hours, washed, and left untreated (solid black lines) or treated with 300 nM DG alone (left, solid red lines), or 3 μM AD alone (right, solid red lines), rifampin alone (dashed black lines) or the combination of DG (left) or AD (right) and rifampin (dashed red lines). Rifampin at 0.5 μM. Cells were lysed at the indicated times for determination of CFU. Results are mean ± SD of triplicate wells in a single experiment representative of 2 independent experiments. (D) The effects of ikarugamycin on Mtb viability in infected BMDM. Experiment was carried out as described in panel C except IKA was used at 300 nM. (TIF) [file ppat.1008567.s014.tif]

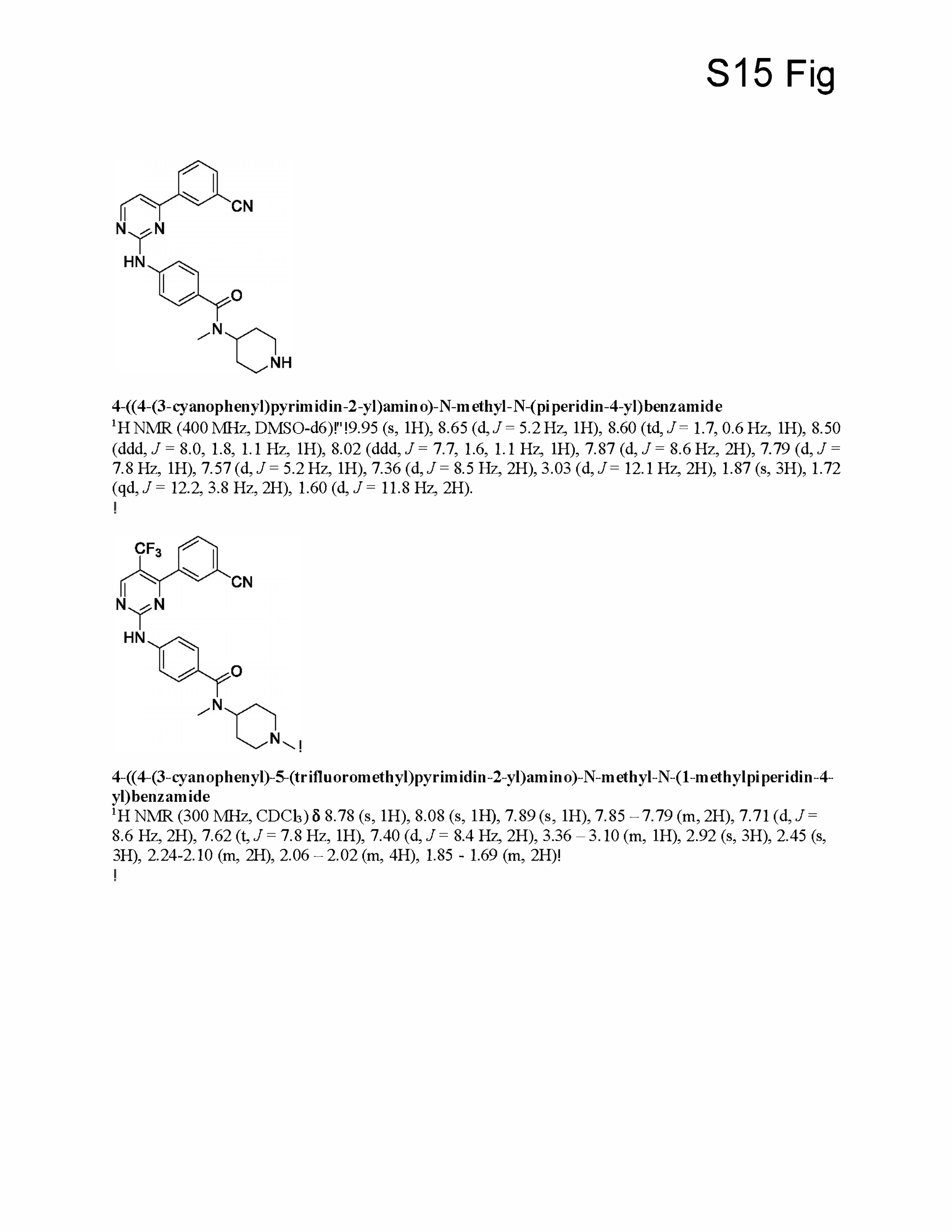

Supplement: S15 Fig — (TIF) [file ppat.1008567.s015.tif]
